# Natural Fish Trap‐Like Nanocage for Label‐Free Capture of Circulating Tumor Cells

**DOI:** 10.1002/advs.202002259

**Published:** 2020-10-15

**Authors:** Wenning Jiang, Lulu Han, Liwei Yang, Ting Xu, Jiabei He, Ruilian Peng, Ziyu Liu, Chong Zhang, Xiaomin Yu, Lingyun Jia

**Affiliations:** ^1^ Liaoning Key Laboratory of Molecular Recognition and Imaging School of Bioengineering Dalian University of Technology Dalian 116023 P. R. China; ^2^ Department of Oncology The Dalian Municipal Central Hospital Affiliated of Dalian Medical University Dalian 116033 P. R. China

**Keywords:** circulating tumor cell, high efficiency, label‐free capture, nanocage, natural pollen

## Abstract

Nanomaterials have achieved several breakthroughs in the capture of circulating tumor cells (CTCs) over the past decades. However, artificial fabrication of label‐free nanomaterials used for high‐efficiency CTC capture is still a challenge. Through billions of years of evolution and natural selection, various complicated and precise hierarchical structures are developed. Here, a novel fish trap‐like “nanocage” structure derived from the natural Chrysanthemum pollen is reported and a nanocage‐featured film for the label‐free capture of CTCs and CTC clusters is constructed. The nanocage‐featured film effectively captures 92% rare cancer cells with a broad spectrum of cancer types, due to the synergistic effect of nanocage‐CTC filopodia matching, high contact area, and strong adhesion force between the cancer cells and the nanocage. Furthermore, the nanocage‐featured film successfully detects CTCs and CTC clusters in 2 or 4 mL blood taken from 21 cancer patients (stages I–IV) suffering from various types of cancers. This novel, abundant, and economical fish trap‐like “nanocage” may provide new perspectives for the application of natural nanomaterials in clinical CTC capture and analysis.

## Graphical abstract

An ingenious fish trap‐like nanocage structure derived from natural chrysanthemum pollen is reported. It precisely matches the structure of circulating tumor cell (CTC) filopodia, providing high contact area and ultrastrong adhesion force to broad‐spectrum cancer cells without any labels. This novel and economical natural material shows superior clinical capture performance of CTCs and CTC clusters from patients with stage I–IV cancer.

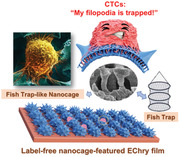

Circulating tumor cells (CTCs) shed from the primary tumor mass into peripheral bloodstream can establish distant metastatic lesions in other organs,^[^ ^1a^ ^]^ which attribute to 90% cancer‐related death.^[^ ^1b^ ^]^ CTC isolation serves as a noninvasive “liquid biopsy”^[^ ^2^ ^]^ that allows the enumeration of rare CTCs from cancer patients, assisting with cancer diagnosis, prognosis monitoring,^[^ ^3^ ^]^ and also helping with the provision of personalized anticancer therapy.^[^ ^3^, ^4^ ^]^ An ideal CTC‐capture material should exhibit several critical characteristics: i) high‐efficiency capture of rare CTCs (as few as one CTC in a billion blood cells), ii) broad‐spectrum capture performance of heterogeneous CTCs, and iii) selective capture of CTCs other than blood cells such as peripheral blood mononuclear cells (PBMCs). The first reported label‐free CTC capture substrate was nanorough glass, which showed a capture yield of 80%.^[^ ^5a^ ^]^

During the past decades, more artificial nanostructured substrates^[^ ^5b^ ^]^ (nanoparticles,^[^ ^5c–e^ ^]^ nanowires,^[^ ^5f–k^ ^]^ nanotubes,^[^ ^5l^ ^]^ and nanofibers^[^ ^5m^ ^]^) and nanostructure‐embedded microfluidic chips^[^ ^6a–c^ ^]^ have achieved several breakthroughs in the capture of CTCs.^[^ ^6d^ ^]^ Size and shape‐matched nanometer‐scale topography enhances the interactions between the materials and the target cells, by promoting the extension of cell filopodia, increasing the contact between filopodia tip and the material, and eventually facilitate cell attachment.^[^ ^7a^ ^]^ However, the interactions between the cell filopodia and the current nanomaterials are still not strong enough.^[^ ^7b^ ^]^ To further enhance the interactions between the substrates and CTCs, hierarchical nanomaterials consisting of nanoscale and microscale components are emerging (i.e., cell‐replica surface,^[^ ^8b^ ^]^ rose‐replica surface,^[^ ^8c^ ^]^ fractal nanostructures,^[^ ^8^, ^9^ ^]^ TiO_2_ nanosisal‐like substrate,^[^ ^9b,c^ ^]^ and nanograss interface^[^ ^9d^ ^]^), which have shown unique advantages of synergistic interactions with CTCs. However, the capture efficiencies of the nonmodified hierarchical nanostructured substrates are still not satisfactory (≈70%).^[^ ^8^, ^9^ ^]^ To increase the isolation efficiency to 90%, these nanomaterials need to be further labeled with active molecules (antibodies, polypeptides, or DNA aptamers) that can recognize the receptors on the cancer cell membrane, consequently increasing the bonding affinity between CTCs and the materials.^[^ ^9^ ^]^ However, due to the heterogeneous expressions of biomarkers on the surface of CTCs,^[^ ^10^ ^]^ this label‐dependent isolation method inevitably misses out certain CTC subpopulations, leading to a false‐negative result.^[^ ^11^ ^]^ Employing a cocktail of antibodies helps isolate a broad spectrum of CTCs,^[^ ^12^ ^]^ but the time‐consuming process, expensive costs, and higher risks of deactivation remain the obstacles limiting its application at the clinical level. Thus, there is an urgent need to develop a high‐efficiency and label‐free nanomaterial for the capture of broad‐spectrum CTCs.

Natural pollen is composed of a carbon‐skeletal outer exine with hierarchical sculptures.^[^ ^13^ ^]^ Up to date, pollens have been widely utilized as a biotemplate and carbon‐rich precursors to produce high surface/volume ratio hollow microspheres, which are applied in catalysis,^[^ ^14^ ^]^ sensing,^[^ ^15^ ^]^ drug delivery,^[^ ^16^ ^]^ and photo‐electrochemistry.^[^ ^17^ ^]^ In addition, the structural characteristics of natural pollen exine have been optimized by natural selection over billions of years, possessing unique, abundant and distinctive micro/nanoscale structures, which serve specific functions in regulating cell behavior. However, these unique natural pollen‐based nanostructures have not been used in cell capture and analysis, mainly due to their autofluorescence^[^ ^18^ ^]^ and difficulty in film fabrication as bulk materials. Herein, we firstly report a fish trap‐like “nanocage” structure derived from natural chrysanthemum (*Chrysanthemum morifolium* Ramat) pollen (Chry pollen), which is capable of inducing and trapping the filopodia of cancer cells, while preventing the anchored filopodia from retracting, leading to the high‐efficiency and label‐free CTC isolation.

The Chry pollen diameter is 22 µm and decorated with sub‐micrometer‐scaled spine (3 µm in length, 255 nm in tip diameter, and a space of 5 µm between adjacent spines) (**Figure** **1**a; Table S1, Supporting Information). After treated with acetone and sodium hydroxide for removal of the surface‐covered fatty coating (Figure 1b), the pollens were further etched with concentrated H_2_SO_4_ for 24 h to make the exterior nanocage exposed adequately (Figure 1c; Figure S1, Supporting Information) and also to quench the autofluorescence of the pollens (Figure S2, Supporting Information) via carbonification and oxidation reactions (Figures S3 and S4, Supporting Information). Eventually, a great number of nanocages as exine sculpture emerged on the exterior surface of the H_2_SO_4_‐etched chrythansemum pollens (EChry pollens). Similar to a fish trap consisting of fish size‐matched mesh, steel wire frame and internal cavity, the nanocage of EChry pollens featured filopodia size‐matched surficial entrances (175 nm in average aperture), internal braced frames (117 nm in average diameter and 525 nm in average height), and interconnected fish trap‐like cavities (166 nm in average spacing; Figure 1d; Table S2, Supporting Information), as revealed by cross‐sectional scanning electron microscopy (SEM) and transmission electron microscopy (TEM) micrographs.

**Figure 1:**
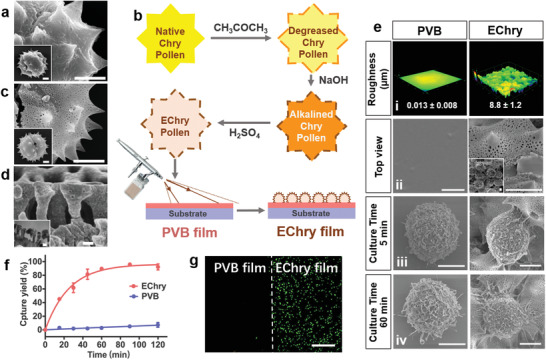
Preparation, characterization and MCF‐7 cell capture performance of nanocage and nanocage‐featured EChry film. a) SEM images of native Chry pollen (scale bar = 5 µm). b) Preparation of EChry pollen and EChry film. c) SEM images of EChry pollen (scale bar = 5 µm). d) Cross‐sectional SEM image of EChry pollen. Inset is a TEM image of the ultrathin section of EChry pollen (scale bar = 100 nm). e) Laser scanning confocal microscope topographical i) and SEM images ii) of PVB and EChry films, and the representative SEM images of MCF‐7 cell captured on the PVB and EChry films after 5 min iii) and 60 min iv) incubation (scale bar = 5 µm). Inset is a zoomed‐out SEM image of the EChry film. f) Dynamic capture yields of MCF‐7 cells on the PVB and EChry films in culture medium (mean ± SD, *n* = 5). g) Fluorescein diacetate‐stained MCF‐7 cells captured on the flat PVB film (left) and the EChry film (right) (scale bar = 200 µm).

To fabricate a nanocage‐featured film, the EChry pollens were suspended in ethanol, and then uniformly spray‐coated on a precoated polyvinyl butyral film (PVB film) via the atomization of the airbrush (Figure 1b). PVB film with a thickness of 3.1 ± 0.3 µm was used as an adhesive layer. When the PVB film contacted with the spraying EChry pollen–ethanol solution, it was first dissolved and then attached to the EChry pollen. After drying, the embedded EChry pollens stably attached to the PVB film (EChry film) and tolerated peeling tests carried out with a 3M Scotch adhesive tape (Figure S5a, Supporting Information) or with converted reciprocal motion, without significantly shedding (Movie S1, Supporting Information). Moreover, this facile method was surface‐independent and applicable to different material surfaces (i.e., inorganic substance (glass), metal (coin), and polymer (silicone tube)) on a large scale regardless of composition and shape (Figure S5b, Supporting Information). Additionally, the resulting EChry film still retained the original nanocage structures of the EChry pollens without the coverage of PVB (Figure 1e).

We evaluated the cancer cell‐capture performance of the EChry film by incubation with a suspension of MCF‐7 cells (breast cancer cells) in the culture medium. To suppress the interferences of the surface chemical properties to cancer cell capture,^[^ ^19^ ^]^ all of the surfaces used for the following cell experiments were blocked with 1% (w/v) inert bovine serum albumin. The correlations between incubation time and the cell‐capture efficiency of EChry film were plotted in Figure 1f. Within the first 5 min of incubation, the initial filopodia of MCF‐7 cells have already aligned and some even anchored to the nanocage entrance (Figure 1e). This result suggests that the MCF‐7 cells could contact and then sense the nanosized entrance of nanocage, inducing the formation of filopodia within a few minutes, which is consistent with the previous reports that the nanostructures enhanced the topographical interactions between cell and substrate.^[^ ^20^ ^]^ After 60 min of incubation, the cell‐capture efficiency of EChry film was significantly higher than that of the naked plat PVB film (Figure 1g; Figure S6, Supporting Information) with the maximum capture yield reaching 91% ± 2% (Figure 1f), which surpasses the previously reported capture yield of the label‐free nanostructured substrates.^[^ ^5a^ ^]^ In addition, the label‐free EChry film showed no significant difference compared with the anti‐epithelial cell adhesion molecule (anti‐EpCAM) antibody modified one in the capture performance of MCF‐7 cells (Figure S7, Supporting Information), proving the excellent cancer cell‐capture performance of EChry film.

The EChry film features a three‐level hierarchical structure, including microsphere, sub‐microspine, and nanocage structure. To rule out the influences of the microsphere and sub‐microspines on cell capture, we fabricated films with smooth PS spheres (20 µm in diameter, similar to that of Chry pollens, named as PS film) and spine‐containing EChry particles (nanocage blocked with PVB solution, named as EChry‐bl film) using the same spray‐coating method to create nanocage‐free films (Figure S8, Supporting Information). Within 60 min of incubation, the capture yields of MCF‐7 cells on the PS film and EChry‐bl films were 24% and 36% (Figure S9, Supporting Information), respectively, indicating that the microsphere and sub‐microspine structures improved the capture yield only to a limited extent. Thus, the nanocage structure of EChry pollen plays a crucial role in the capture of cancer cells.

Because the cell‐capture performance of biomaterials is highly related to cell‐material interactions, the interactions between the nanocage and MCF‐7 cells were investigated using a confocal laser scanning microscope (CLSM) and TEM, while PBMCs were chosen as a control to confirm the selective matching effects of nanocage on cancer cell filopodia. We found that the process of EChry nanocage trapped MCF‐7 cell filopodia with the following three steps. i) The cancer cells contacted and sensed the surficial nanosized entrance while the initial filopodia were induced to align. And after 60 min, the MCF‐7 cells captured on the EChry film possessed numerous filopodia with an average diameter of 131 ± 38 nm (Figure 1e; Figure S10, Supporting Information). Immunofluorescent staining images (**Figure** **2**a) further visualized that abundant actin‐rich filopodia reorganized and protruded from the MCF‐7 cells and spread all over the surface of the nanocage, with mature vinculin‐containing focal adhesions formed on the peripheral region (Figure S11, Supporting Information), implying that mechanical links were built between the intracellular actin bundles and the surface of EChry nanocage just within 60 min.^[^ ^5a^ ^]^ Meanwhile, the captured cells exhibited the lowest nucleolus height (13 µm ± 2 µm) than the cells captured on PVB, PS and EChry‐bl film (Figure S12, Supporting Information), demonstrating the better spreading and intimate connection between the cell filopodia and nanocage.^[^ ^21^ ^]^ ii) Next, as filopodial tips play a sensory role in searching for suitable anchorage sites to guide the development of filopodial,^[^ ^22^ ^]^ they further anchored into the entrance of nanocage whose size is slightly larger than the filopodia (average diameter of the entrance is 175 nm; Table S2 and Figure S10a, Supporting Information). Notably, after anchoring into the nanocage entrance, the filopodia were guided along with the vertical braced frame which served as nanobridge^[^ ^23^ ^]^ and extended into the nanocage cavity inside (Figure 2b). iii) Finally, the filopodia grew deeper into the bottom of the nanocage, being sandwiched between the braced frames (Figure 2b), while some of that further extended through the interconnected cavities to the neighboring nanocage. Moreover, the filopodia of MCF‐7 cells even largely deformed and wrapped around the internal frame suggesting a tight attachment (Figure 2b,c). In contrast, PBMCs displayed shorter and thicker (diameter of 268 ± 73 nm) microvillus less frequently (Figure S10b–d, Supporting Information), thereby it was harder for the PBMCs to insert into the nanocage (Figure 2d,e). As a result, the EChry film exhibited a remarkably low PBMC‐capture yield (≈8%). These data demonstrate that the nanocage prefers to the filopodia of cancer cells, which is more matched with the nanocage than that of PBMCs (Movie S2, Supporting Information).

**Figure 2:**
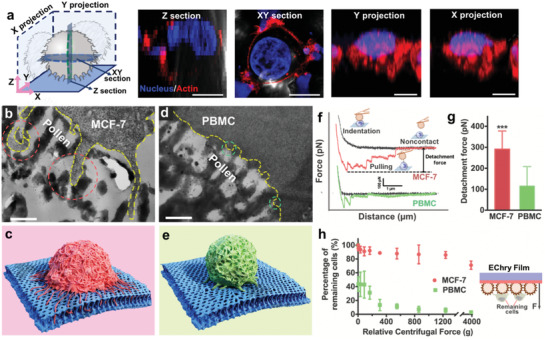
Preferential interactions of nanocage to MCF‐7 cancer cell over PBMC. a) 3D CLSM analysis of a single cell (left) and fluorescence images of a representative MCF‐7 cell captured on the EChry film observed from the reconstructed Z section, the cross‐sectional XY section, and the orthogonal X and Y projection planes (scale bar = 10 µm). F‐actin was stained with rhodamine‐conjugated phalloidin (red), and the nucleus was stained with DAPI (blue). b) TEM image of ultrathin section and c) schematic illustration of a captured MCF‐7 cell on EChry film with spread filopodia (red dotted circles) stuck into the nanocage (scale bar = 500 nm). Yellow dotted lines sketch the contour of the cell. d) TEM image of ultrathin section and e) schematic illustration of an adhered PBMC with short embedded microvillus (green dotted circles). f) Representative AFM force–distance curves and g) detachment forces required of separating the EChry pollen‐bound AFM tip from the surface of a single live MCF‐7 cell and PBMC, respectively (mean ± SD, *n* = 9). ***: *p* < 0.001. h) Percentage of the remaining MCF‐7 cells and the PBMCs on EChry film under dethatched centrifugal forces (left, mean ± SD, *n* = 5) in the detachment assay (right).

To further illuminate the cancer cell filopodia‐nanocage matching effect, we then assessed the effective contact area between the filopodia and the nanocage. On previously reported bed‐of‐nails nanostructures, such as silicon nanocolumn,^[^ ^24a^ ^]^ nanowires,^[^ ^24b^ ^]^ and TiO_2_ nanorod arrays,^[^ ^24c^ ^]^ cells just “float” on the substrates and make contact using their tips,^[^ ^10^, ^24^ ^]^ with a contact area of 0.008–0.018 µm^2^ per filopodia.^[^ ^25^ ^]^ Whereas on the fish trap‐like nanocage with internal braced frames and interconnected cavity, the elongate filopodia of cancer cells can make contact with the material from root to tip (Figure 2b), resulting in an effective contact area of 0.1–0.2 µm^2^ per filopodia (Figure S13, Supporting Information), which enhanced 10 times than that of the bed‐of‐nails nanostructures. To further confirm the necessity of the interconnective nanocage structure to make effective contact, we used nanopore‐containing PS spheres (with a similar diameter to EChry pollen) as control. The spheres have similar nanoscale entrances as the nanocage but do not present the internal braced frame or interconnected cavity (Figure S14a, Supporting Information). We found this nanopore‐containing PS film prepared using the same spray coating exhibited much lower CTC‐capture yield (≈19%; Figure S14b, Supporting Information), indicating the internal braced frame and interconnected cavity were essential in providing sufficient contact between the filopodia of the cancer cells and the nanocage.

Furthermore, the sufficient contact between cancer cell filopodia and nanocage leads to high contact force, which prevents the filopodia from withdrawing from the fish trap‐like nanocage. Herein, we used atomic force microscopy (AFM) to measure the contact forces between EChry pollen and a single cell in the initial contact process (within 15 s; Figure 2f). Retraction force–distance curves showed that a detachment force of 291 ± 86 pN was required to separate the EChry pollen‐bound AFM tip from the surface of a single living MCF‐7 cell (Figure 2g), notably higher than that required to separate the same AFM tip from a PBMC. This indicated that a stronger contact force between MCF‐7 filopodia and the nanocage had been established in the initial cell adhesion phase. To obtain a better understanding of this adhesion force of cancer cell population on the nanocage, we applied a reversed centrifugation force^[^ ^26^ ^]^ exerted on the cell‐attached EChry films after 60 min of cell capture (Figure 2h). The data indicated that about 86% of MCF‐7 cells remained on the EChry film after centrifugation with 1238 × *g*, and ≈71% of the cells still maintained on the film even when the centrifugal force was increased to 3834 × *g* (the reported maximum centrifugal force used in centrifugation assay). The calculated adhesion force between MCF‐7 cells and the nanocage was above 23 nN, which is ≈100‐fold larger than the reported adhesion force used to detach the cells making contact only at the tips of the filopodia.^[^ ^7^, ^25^, ^27^ ^]^ Besides that, 80% of various cancer cells (such as A431 and Hela cells) remained on the EChry films after centrifugation with 1238 × *g* (Figure S15, Supporting Information). These findings suggest the nanocage provided ultrastrong cell‐material adhesion for the broad‐spectrum cancer cells. However, less than 6% of PBMCs and 7% of preadhered human promyelocytic leukemia cells (HL‐60 cells) remained on the films at 1238 × *g*, indicating that the nanocage‐featured EChry film displayed tighter binding toward cancer cells than leukocytes, and this could be pivotal to the superior capture performance of CTCs by the EChry film.

From the above results, the nanocage, which is larger than the CTC filopodia, allows the filopodia to anchor after entering the surficial entrance and to further extend through the interconnected cavities with high contact area, resulting in the entrapment of CTC filopodia with ultrastrong adhesion force. The mechanism of effective capture of cancer cells by the nanocage structure is concluded to be the synergistic effect of nanostructure matching, high contact area and ultrastrong adhesion force between the nanocage and the filopodia of cancer cells. To further verify the aforementioned mechanism, we first change the pollen species to regulate the sizes of nanostructures to validate the uniqueness of the nanocage of EChry pollen. H_2_SO_4_‐etched pine pollen (EPine*, Pinus sylvestris L*.) and rape pollen (ERape, *Brassica napus L*.) were chosen as controls owing to their similar frame sculpture but different sizes compared to the EChry pollen. The EPine pollen featured smaller surficial entrance (≈72 nm in average diameter) which prevented filopodia from penetrating and coming into contact with the internal area of the pollen (Figure S16, Supporting Information). Meanwhile, the ERape pollen featured larger surficial entrance (≈540 nm in average diameter) that effectively induced filopodia inserting, nevertheless, the large interspace of ERape pollen sculpture resulted in smaller surface areas, thus decreased the contact area and adhesion force (Figure S17, Supporting Information) between the filopodia and the material. Finally, both EPine and ERape featured‐films showed a lower capture yield (40–70%; Figures S16 and S17, Supporting Information) as compared to the EChry featured film. In consequence, the delicate nanocage structures of EChry pollen: the surficial entrance (≈175 nm), the interconnective cavity (≈166 nm), and the internal frame (≈117 nm) are all suitable for the cell filopodia to sense and to extend, simultaneously maximizing the contact area for the filopodia to anchor and to adhere. This result is also consistent with the previous report that 140–200 nm spacing suits the efficient and specific inserting of cancer cell filopodia.^[^ ^28^ ^]^

Next, to prove the importance of filopodia in the cell capture mechanism of EChry nanocage, we also evaluated capture yields of the other two cell types on the EChry film within 60 min of incubation. The filopodia‐rich mesenchymal stem cells (MSCs) were used as a positive control and the filopodia‐poor HL‐60 cells were used as a negative control. The results showed that the nanocage structure of EChry pollen displayed a high capture performance (≈95%) for filopodia‐rich MSCs (Figure S18a,b, Supporting Information) and a remarkably low capture performance (≈7%) for filopodia‐poor HL‐60 cells (Figure S18c, Supporting Information) which verifies that the superior cell‐capture performance of the EChry film highly relies on the trapping of cell filopodia. Inspired by this mechanism, the nanocage‐featured film shows potential as a biomaterial for the capture and support of filopodia‐rich cells while discriminating filopodia‐poor cells (such as PBMCs).

To quantificationally examine the rare cancer cell‐capture performance of nanocage, we further loaded 10–4000 cancer cells into 1 mL of 10^6^ PBMC suspension (**Figure** **3**a), and regarded the mixture as imitated blood samples. About 92% ± 2% of the prestained MCF‐7 cells in the PBMC suspension were captured by the EChry film (Figure 3b), and the correlation between the number of captured MCF‐7 cells and that of the cells loaded in the suspension was almost linear (Figure 3c; *R* ^2^ = 0.99). Furthermore, the EChry film was capable of more than tenfold enrichment of the rare cancer cells from massive background PBMCs (from initial 10–4000 MCF‐7 cells/10^6^ PBMCs to 9–3689 MCF‐7 cells/8 × 10^4^ PBMCs). Based on the higher adhesion force of MCF‐7 cells over PBMCs, the reversed centrifugation method was used to improve the capture purity (Figure S19, Supporting Information), but it was not further used for the precise CTC counting, due to the loss of ≈13% captured cancer cells after centrifugation with 1238 × *g*. Notably, the nanocage of the EChry pollen could capture a broad spectrum of cancer cell types, including A431 (epithelial cell adhesion molecules+, EpCAM+), Hela (EpCAM−), A549 (EpCAM−), and HepG2 (EpCAM−), from the PBMC suspension, giving a capture yield as high as 93% (Figure 3d). Compared with the reported anti‐EpCAM antibody‐modified hierarchical nanostructured substrates,^[^ ^9^ ^]^ the nanocage‐featured film demonstrates a superior and broad‐spectrum CTC capture performance, but without any functional modification (**Table** **1**).^[34‐37]^

**Figure 3:**
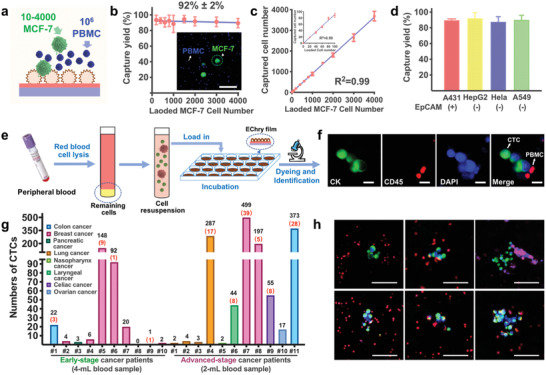
Broad‐spectrum and high‐efficiency capture of CTCs and CTC clusters on nanocage‐featured EChry film. a) Schematic illustration of the rare cancer cell‐capture experiment. b) Capture yields of MCF‐7 cells from PBMCs on EChry film (mean ± SD, *n* = 5). Inset in (b) shows a representative immunofluorescence image of captured cells from 10^6^ PBMC suspensions containing 4000 MCF‐7 cells (scale bar = 100 µm). c) The number of MCF‐7 cells captured on EChry film as a function of loaded cell number (mean ± SD, *n* = 5). d) Capture yields of various cancer cells on EChry film (mean ± SD, *n* = 5). e) Schematic illustration of the procedures of blood sample pretreatment and CTCs capture from patients’ peripheral blood using EChry films. f) Immunofluorescence images of captured CTCs from cancer patients’ blood using a three‐color immunocytochemistry method. CTCs were distinctly stained with anti‐CK (green) and DAPI (blue), and the PBMCs were stained with anti‐CD45 (red) and DAPI (blue) (scale bar = 20 µm). g) Quantification of CTCs and CTC clusters from 4 mL blood samples of early‐stage cancer patients (stages I and II) and 2 mL blood samples of advanced‐stage cancer patients (stages III and IV). The number of captured CTC clusters is marked in red. h) Representative merged immunofluorescence images of the CTC clusters captured from cancer patients’ blood (scale bar = 100 µm).

**Table 1:** Capture yield comparison between the reported hierarchical nanostructured substrates and the nanocage‐featured EChry film

	Capture yield [%]			
		With anti‐EpCAM modified		
Hierarchical nanostructured substrates	Without anti‐EpCAM modified^a)^	EpCAM (+)^b)^	EpCAM (−)^c)^	Ref.
ZnO nanograss interface		90%	22%	^[^ ^9d^ ^]^
Si NW‐decorated frosted slides		85%	20%	^[^ ^9e^ ^]^
Au NC‐coated Si NWs	40%	88%		^[^ ^9h^ ^]^
TiO_2_/MnO_2_/FTO nanorod arrays		92%		^[^ ^9c^ ^]^
Multiscale TiO_2_ nanorod array	70%	93%		^[^ ^9b^ ^]^
Fractal nanowire arrays	40%	89%	10%	^[^ ^9a^ ^]^
Fractal gold nanostructures	21%	62%	2%	^[^ ^8a^ ^]^
Flowerlike HZnPNS	27%	90%	28%	^[^ ^9f^ ^]^
Cancer cell‐replica surface	70%	90%	70%	^[^ ^34^ ^]^
Rose petals‐replica surface	10%	95%	20%	^[^ ^8c^ ^]^
Pollen‐replica surface		72%	1%	^[^ ^35^ ^]^
leukocyte‐inspired particles (LIPs)	22%	62%	6%	^[^ ^9g^ ^]^
Lotus leaf‐like biointerfaces		74%	5%	^[^ ^36^ ^]^
rGO/ZnO foam	11%	58%		^[^ ^37^ ^]^
Nanocage‐featured EChry film	92% (label‐free)	93% (label‐free)	92% (label‐free)	This work

^a)^ Capture yields of nanostructured substrates without anti‐EpCAM antibody modified;

^b)^ Capture yields of EpCAM‐expression positive cells using the indicated anti‐EpCAM antibody modified nanostructured substrates;

^c)^ Capture yields of EpCAM‐expression negative cells using the indicated anti‐EpCAM antibody modified nanostructured substrates.

The cytocompatibility of the EChry film was verified to guarantee downstream molecular biological analysis for captured cancer cells. Various captured cancer cells (Hela, A431, and MCF‐7) on the EChry films presented up to 97% viability (Figure S20, Supporting Information), and the number of adherent MCF‐7 cells increased by 1.8 times after 48 h of culture, indicating good viability of the captured cancer cells on the EChry films.

Furthermore, this nanocage‐featured label‐free EChry film was applied to capture CTCs from blood red cell lysed cancer patients’ blood samples (Figure 3e). Herein, rare cancer cells in lysed blood samples had a similar capture yield as those in PBMC suspension (Figure S21, Supporting Information). CTCs were defined as nucleated cells expressing cytokeratin and lacking CD45 (Figure 3f).^[^ ^29^ ^]^ Using this CTC capture film, both single individual CTCs and CTC clusters (a group of more than two tumor cells) were successfully isolated 2–499 CTCs from 2 mL blood samples taken from 11 patients with different kinds of advanced cancer (Figure 3d; Table S3, Supporting Information), including lung cancer, nasopharyngeal cancer, laryngeal cancer, nasopharyngeal cancer, ovarian cancer, breast cancer, and colorectal cancer with a high detection rate of 100% (Figure 3g). Moreover, 4 mL blood samples of 10 early‐stage cancer patients were examined with a positive detection rate of 90% (Table S4, Supporting Information), indicating high detecting sensitivity of our nanocage‐featured EChry film. In contrast, no CTC was detected from the 4 mL blood samples of seven noncancer subjects undergoing routine health checkups, reinforcing the reliability of our novel CTC‐capture method for potential clinical practices (Table S4, Supporting Information).

Notably, massive CTC clusters were detected in more than half of the advanced cancer patients (6/11) (Figure 3h; Figure S22, Supporting Information). In specific, 39 CTC clusters were detected in one breast cancer patient with postoperative recurrent‐bone metastasis. Compared with individual CTCs, CTC clusters formed by about 3% of all CTCs are rarer,^[^ ^30^ ^]^ and have higher metastatic potentials.^[^ ^31^ ^]^ Thus, the quantification and analysis of CTC clusters would provide more important prognostic values.^[^ ^32^ ^]^ However, the capture of CTC clusters is more difficult because of their smaller surface‐area‐to‐volume ratio, which minimizes the interactions between CTC clusters and the surface of the capturing material. Due to the effect of structure‐matching between CTC filopodia and nanocage, the nanocage‐featured EChry films that we constructed were sufficiently sensitive to detect rare CTC clusters and to distinguish the clusters from background PBMCs without extra bioactive molecules. Furthermore, the static CTC‐capture method shows a lower stress force compared with the microfluidic systems, which is beneficial to preserve the integrity of CTC clusters.^[^ ^33^ ^]^ Although the EChry film shows outstanding capture performance of CTCs and CTC clusters, the epithelial‐to‐mesenchymal transition (EMT) of CTCs during the process of shedding and metastasis may lead to changes in cell morphology and the adhesion signaling pathways,^[^ ^33c^ ^]^ the corresponding impact on the capture performance of EChry film would be further investigated in future research.

In summary, we report a novel fish trap‐like nanocage structure derived from natural Chry pollens and fabricate an effective nanocage‐featured film for CTCs capture which demonstrates several advantages: first, our nanocage‐featured film is the first reported label‐free nanostructured film to allow the capture of broad‐spectrum cancer cells with a capture yield up to 92%. Second, the natural nanocage structure uniquely prefers cancer cells to PBMCs due to the nanocage‐filopodia matching effect. Third, in addition to the individual CTCs, the nanocage‐featured film also isolates rarer CTC clusters. Most importantly, compared with the artificial nanomaterials, nanocage derived from the natural pollen is delicately structured, abundantly available and cost‐effective. Thus, the natural nanocage‐featured pollen and film can be considered as new promising nano‐biomaterials offering great perspectives for the CTCs and CTC clusters isolation in future clinical applications.

## Conflict of Interest

The authors declare no conflict of interest.

## Supporting information

Supporting Information

Supplemental Movie 1

Supplemental Movie 2
