# Supplementary material for: Natural Fish Trap‐Like Nanocage for Label‐Free Capture of Circulating Tumor Cells
Source: Adv Sci (Weinh). 2020 Oct 15;7(22):2002259. doi: 10.1002/advs.202002259 (PMC7675191; doi:10.1002/advs.202002259)
Supplement: Supplementary file 1 — Supporting Information [file ADVS-7-2002259-s001.pdf]

WILEY-VCH

© 2020 Wiley-VCH GmbH

ADVANCED SCIENCE
Open Access

Supporting Information

for *Adv. Sci.*, DOI: 10.1002/advs.advs202002259

Natural Fish Trap-Like Nanocage for Label-Free Capture of
Circulating Tumor Cells

Wenning Jiang, Lulu Han, Liwei Yang, Ting Xu, Jiabei He, Ruilian Peng, Ziyu Liu, Chong Zhang, Xiaomin Yu, and Lingyun Jia**

Supporting Information

Natural Fish Trap-Like Nanocage for Label-Free Capture of Circulating Tumor Cells

Wenning Jiang, Lulu Han, Liwei Yang, Ting Xu, Jiabei He, Ruilian Peng, Ziyu Liu, Chong Zhang, Xiaomin Yu, and Lingyun Jia**

Experimental Section

Material Formulation:

Chrysanthemum pollens were purchased from Yifeng Apiculture (Changge, China). Rape pollens were purchased from Wutaishan Apiculture (Xinzhou, China). Pine pollens were purchased from Yier Apiculture (Taian, China). Polystyrene (PS) particles were purchased from Knowledge & Benefit Sphere technology (Suzhou, China). Glutaraldehyde (2.5%) was purchased from Solarbio Biotechnology (Beijing, China). Paraformaldehyde (PFA), fluorescein diacetate (FDA) and polyvinyl butyral (PVB) were bought from J&K Chemical (Beijing, China). 4’,6-diamidino-2-phenylindole (DAPI), 3,3′-dioctadecyloxacarbocyanine perchlorate (DiO) and phalloidin were purchased from Yeasen Biotechnology (Shanghai, China). Fetal bovine serum (FBS), biotinylated bovine serum albumin (biotin-BSA), streptavidin (SA), penicillin-streptomycin, streptavidin, trypsin ethylene diaminetetraacetic acid (0.25%), high-glucose Dulbecco's Modified Eagle's Medium (DMEM) with L-glutamine, Roswell Park Memorial Institute (RPMI) 1640 medium with L-glutamine, Iscove’s Modified Dubecco’s Medium (IMDM) with L-glutamine and pan-cytokeratin monoclonal antibody (AE1/AE3)-Alexa Fluor 488 were purchased

from Thermo Fisher Scientific (Waltham, MA). Anti-EpCAM antibody was purchased from Abcam (Cambridge, MA). CD45 polyclonal antibody and donkey anti-rabbit IgG-Alexa Fluor 546 were purchased from Invitrogen (Carlsbad, CA). Rabbit anti-vinculin primary antibody was purchased from Bioss (Beijing, China). Red blood cell lysis buffer and Alexa Fluor 488-conjugated goat antirabbit IgG secondary antibody was purchased from Solarbio Life Sciences (Beijing, China). Phosphate buffer saline (PBS, pH 7.4) was bought from GE Healthcare Life Sciences (Logan, UT). All other reagents were of analytical grade. The water used in all experiments was obtained from an ultrapure water purification system (Millipore, U.S.A.) with a resistivity of 18.2 MΩ.cm. All aqueous solutions were filtered with 220 nm diameter membranes before use.

Preparation of H_2SO_4-etched Chrysanthemum Pollens:

Natural Chrysanthemum pollens were defatted by suspending in acetone and stirred under reflux for 6 h. After that, the defatted pollens were filtered and subjected to alkaline lysis in fresh sodium hydroxide solution (6%, w/v) with stirring under reflux for 12 h. The suspension was filtered and washed with water and ethanol, and then dried overnight. The pollens were next suspended in 1 L of concentrated sulfuric acid (98%) inside a flask and gently mixed to form a homogeneous suspension. This was further stirred under reflux for 24 h. After that, the pollens were collected by centrifugation and washed extensively with water and ethanol. Finally, the resulting hollow H_2SO_4-etched pollens (designated as EChry pollens) were dried at room temperature for 48 h in a vacuum dryer. After complete drying, the samples were

sputter-coated with gold, observed and photographed with a NOVA NanoSEM 450 (FEI, U.S.A.). SEM images of the EChry pollens and nanocage were taken and the images were used for the quantitative and morphological analysis of the pollens and nanocage using the software of Nanomeasurer.

Surface Spray-coating of EChry films:

PVB film was fabricated on a flat glass substrate by applying a PVB ethanol solution (10%, v/v) via spin-coating at a rotation rate of 4000 rev. min^{-1} for 30 s in air, or via spraying the same PVB solution on an irregular or curved substrate, such as silicone tube, and metal coin. EChry pollens–suspended ethanol solution (25 mg mL^{-1}) was then sprayed over the PVB film using an airbrush (U-STAR, A-119) at a nozzle height of 10 cm, a carrier gas pressure of 30 psi and a spraying time of 10 s. The obtained EChry PVB film was purged with N_2 to remove the excess pollens and then blown-dried. After complete drying, the film was sputter-coated with gold, observed and photographed with a NOVA NanoSEM 450 (FEI, U.S.A.).

Cell Culture:

MCF-7, A431, Hela, and A549 cells (Shanghai Institute for Biological Sciences, Chinese Academy of Science) were maintained in a growth medium containing high-glucose DMEM supplemented with 10% (v/v) FBS and 1% (v/v) penicillin-streptomycin. HepG2 cells (Shanghai Institute for Biological Sciences, Chinese Academy of Science) were cultured in RPMI-1640 supplemented with 10% (v/v) FBS and 1% (v/v) penicillin-streptomycin. HL-60 cells (Shanghai Institute for Biological Sciences, Chinese Academy of Science) were cultured in IMDM supplemented with 20% (v/v) FBS and 1% (v/v) penicillin-streptomycin. Mouse

mesenchymal stem cells (MSC cells) were obtained as a gift from Prof. Huanan Wang (Biomat & Tissue Engn Lab, School of Bioengineering, Dalian University of Technology). The cells were maintained at 37°C under 100% humidity and 5% CO_2. Fresh 0.25% trypsin-EDTA in PBS was used to resuspend cells.

Cell capture experiments performed in culture medium:

EChry films (1 × 1 cm^2) were incubated with 1% BSA for 1 h at room temperature and then placed in a 24-well plate (1 piece per well) loaded with cell suspension (1 mL per well, containing 10^5 cells). After incubation at 37 °C and 5% CO_2 for a certain time, the cell suspension was removed and the films were gently rinsed with PBS for three times. After fixed with 4% PFA and gently rinsed with PBS, the captured cells were incubated with PBS containing 10 μg mL^{-1} DAPI for 15 min, and then photographed and the number of captured cells was counted from the image (10 × objective) using Image-pro-plus. Cell capture yield was calculated using the number of cells per unit area as the following formula:

Capture yield = $\frac{x}{\mathrm{N}}$ 100%

where x is the practical number of capture cells per image, and N is the theoretical cell number per image, calculated as 1000 in this experiment.

To give an average captured cell number of x, twenty-five fluorescent images (10 × objective) were uniformly taken of the whole area of cell-capture films (1 × 1 cm^2) without any subjective intention. Five parallel experiments (films were different batches) were carried out to give an error bar for each cell-capture yield.

SEM analysis of Captured cells:

Cells captured on the films were rinsed with PBS and fixed with 2.5% glutaraldehyde for 2 h at room temperature. The cells were then dehydrated through a series of ethanol concentrations (30%, 50%, 70%, 80% and 90%), each lasting for 5 min, followed by lasting for 15 min in absolute ethanol for two times. After that, the cells were dried in liquid CO_2 using a supercritical point dryer to maintain the morphology of the captured cells. After complete drying, the samples were sputter-coated with gold, observed and photographed with a NOVA NanoSEM 450 (FEI, U.S.A.). The FE-SEM images were used to directly determine the number of filopodia and their diameters using the software of Nanomeasurer.

TEM Observation:

Cells captured on the EChry pollens were fixed with 2.5% glutaraldehyde at room temperature for 30 min followed by fixation at 4 °C overnight. The sample was centrifuged and rinsed with PBS for three times, and then dehydrated in a graded ethanol series. Subsequently, absolute ethanol (Gold Shield) was exchanged for acetonitrile. This was followed by infiltration with a graded Embed 812 resin series. The resin-block sample was incubated at room temperature for 1.5 h and then baked at 65 °C for 24 h. The resin block was then trimmed and sectioned on a Leica Ultracut ultramicrotome into 70 nm-thickness sections and imaged on a Tecnai F30 (FEI, U.S.A.).

Immunofluorescence Staining of Captured Cells:

The captured cells were treated with 4% PFA for 15 min and permeabilized with 0.1%

Triton X-100 for 10 min. Then the captured cells were incubated with 1% BSA in PBS for 1 h at room temperature and then incubated with 10 μg mL^{-1} rabbit anti-vinculin primary antibody at 4 ^oC for 12 h and 10 μg mL^{-1} Alexa Fluor 488-conjugated goat antirabbit IgG secondary antibody at 37 ^oC for 1 h. Afterward, the cells were subsequently incubated with 10 μg mL^{-1} rhodamine-conjugated phalloidin (in PBS) for 30 min, and then incubated with 10 μg mL^{-1} DAPI (in PBS) for 5 min. Finally, the cells were examined under a confocal laser scanning microscope and images of the cells were recorded and used to determine the heights of nucleus using the software of Image-Pro Plus.

Anti-EpCAM modified assay:

The PVB, PS, EChry-bl and EChry films were separately loaded into a 24-well cell culture plate, each well of which was immersed in 1 mL of biotin-BSA solution (0.5 mg mL^{-1} in PBS) at 37 °C for 2 h and then rinsed with PBS for three times. Afterward, the films were treated with 10 μg mL^{-1} of streptavidin (SA) at 37 °C for 30 min. After rinsing with PBS for three times, 100 μL of biotin-anti-EpCAM (10 μg mL^{-1} in PBS) was added onto the films at 37 °C for 30 min and then rinsed with PBS for three times.

Collecting and Processing Human Blood Specimen:

Blood specimens were drawn from healthy donors. Sodium citrate was added to the blood samples as an anticoagulant. Peripheral blood mononuclear cells (PBMCs) were isolated from the blood using a human mononuclear cell separation kit (Beijing

Solarbio Co. Ltd, China) according to the manufacturer's specifications.

Rare Cell Capture Experiments Performed in Isolated PBMCs:

Prior to cell-capture assays, the targeted MCF-7 cancer cells were pre-labeled with DiO (10 μg mL^{-1} in PBS) for 30 min. The target-number cells (10–4000) were added into the 1-mL PBMC suspension (10^6 mL^{-1}). The mixtures were loaded onto the EChry films (2 cm^2 in round), and incubated at 37 °C and 5% CO_2 for 60 min. After that, the captured cells were rinsed three times with PBS, fixed with 4% PFA for 15 min, and stained with DAPI (10 μg mL^{-1} in PBS) for 30 min, and examined under a fluorescent inverted microscope. The captured MCF-7 cells and PBMCs were distinguished from each other based on their fluorescence: blue (DAPI+) and green (DiO+) for MCF-7 cells, and only blue (DAPI+) in the case of PBMCs. To accurately count the captured cell numbers, more than 16 fluorescent images (0.125 cm^2 per image with 4 × objective) were taken from one EChry film without missing or overlapping any area. The total number of the captured rare cancer cells was the sum of captured cells number on each image. Cell capture yield was calculated using the ratio of the total number of captured cells to loaded cancer cell numbers. Additionally, the accuracy of the rare cell capture yields was double-checked through counting the number of uncaptured cells to further calculate the captured yields with a subtraction method. The difference in capture yields between these two methods should be less than 1%.

Cell Adhesion Strength Measurements:

The abilities of MCF-7, A431, Hela, HL-60 cells and PBMCs to adhere to the EChry films were measured according to the method reported by Reyes' work.[1] Cell suspensions (1 mL per well, containing 10^5 MCF-7, A431 and Hela cells or 10^6 HL-60 cells and PBMCs) were loaded on the EChry films (1 × 1 cm^2) in a 24-well plate (1 piece per well). Following the cell-capture assay, the cells adhered to the films were incubated with 2 μg mL^{-1} FDA in PBS solution for 5 min, and the images of the cells were then taken under an inverted fluorescence microscope (10 × objective). Subsequently, 10-mL tapered plastic centrifuge tubes were filled with PBS, individual films containing the cells were then carefully laid on the bottom of the tubes with the side containing the cells facing downward. The tubes were gradually centrifuged in an Eppendorf centrifuge (5840 R) at 6, 34, 88, 166, 309, 550, 859, 1238, and 3834 $\times g$, each for 5 min at room temperature. After each round of centrifugation, the cells that remained on the film were examined and counted using the same microscope and under the same magnification (10 × objective). The percentage of cells adhered to the film was calculated using the following formula:

Percentage of remained adhesion cells = $\frac{y}{x}$ 100%

where x is the number of cells attached to the EChry films before centrifugation, and y is the number of cells remained on the film after centrifugation. A total of 25 images were taken to present an average value for x and y. Five experiments were carried out in parallel using different batches of films.

The relative centrifugal force imposed on the cells varies according to the

following equation:

$$F_c = (\rho_{cell} - \rho_{medium}) \cdot V_{cell} \cdot \omega^2 \cdot (r_0 + x)$$

where F_C is the relative centrifugal force, ρ_{cell} is the specific density of the cell (1.07 g cm^{-3}), ρ_{medium} is the specific density of the medium (1 g cm^{-3}), V_{cell} is the cell volume (4000 μm^3 for MCF-7 cell, 500 μm^3 for PBMC), r_0 is the radius of rotation (determined by the dimensions of the rotor), and x is the lateral distance from the bottom of the tube to the center of centrifuge. The force value at which 50% of the cells were detached was determined as the population adhesion strength.

AFM Force Measurements:

AFM force measurement (Nanowizard® Ultraspeed, JPK Instruments, Berlin, Germany) was carried out with live MCF-7 cells and PBMCs immobilized on a plate coated with poly-L-lysine (0.1 mg mL^{-1}, incubated overnight before cell immobilized). The measurement was carried out in PBS. The EChry pollen-modified cantilever was then positioned on the center of a cell, and force-distance (F-z) curves were acquired by approaching the cell with the cantilever at 1 μm s^{-1}, and the contact was maintained for 15 s before the cantilever was retracted at the same speed. The maximum indentation force applied was 100 pN. To collect the adhesion force data, force-curves were obtained for at least nine cells from a minimum of three independent experiments, and an average value of the collected force data was obtained with the help of JPK Data Processing software.

Quantification of Capture Purity of Cancer Cells in the Mixture with PBMCs:

Prior to cell capture assays, the targeted MCF-7 cancer cells were labeled with DiO (10 μg mL^{-1} in PBS) for 30 min. 4000 MCF-7 cells and 10^6 PBMCs (1:250) were mixed in 1 mL of cell culture medium, and then loaded onto the EChry film (2 cm^2 in round). After 1 h incubation, the EChry films were centrifugated with 0 × *g*, and 1238 × *g*. The captured cells were treated with 4% PFA for 15 min, and then incubated with DAPI (10 μg mL^{-1} in PBS) for 15 min, examined under the fluorescent inverted microscope. The captured MCF-7 cells and PBMCs were distinguished from each other based on their fluorescence: blue (DAPI+) and green (DiO+) for MCF-7 cells, and only blue (DAPI+) in the case of PBMCs. To accurately count the captured cell numbers, fluorescent images (4 × objective) were taken from the whole EChry film without missing or overlapping any area, and the number of cells was counted from the image using Image-pro-plus.

Cell Viability Test:

Cell viabilities of captured Hela, A431, and MCF-7 cells on the EChry films were investigated using a Live/Dead staining method with fluorescein diacetate/propidium iodide (FDA/PI) dyes. The FDA/PI working solution was composed of 4 μg mL^{-1} FDA solution and 10 μg mL^{-1} PI solution at the volume ratio of 1:1. After the cell-capture experiments, the captured cells were stained by adding 0.5 mL of FDA/PI working solution onto the films for 5 min. Fluorescent images (10 × objective) were taken to evaluate cell viability.

Cell Proliferation Assay:

A total number of 5 × 10^4 mL^{-1} MCF-7 cells were loaded on the EChry films (2 cm^2 in round). After the cell-capture experiments for 60 min, the EChry films with the captured cells (MCF7 cells) were immersed in fresh DMEM medium for long-term culture. After a certain time of incubation (0, 12, 24, and 48 h), the captured cells were stained by 2 μg mL^{-1} FDA solution for 5 min, and fluorescent images (10 × objective) were taken under the fluorescent inverted microscope, and the number of cells was counted from the image using Image-pro-plus.

Cell Capture Experiments Performed in the Patients' Blood:

EDTA-anticoagulated whole blood samples taken from 21 advanced and early-stage cancer patients (including lung cancer, nasopharyngeal cancer, laryngeal cancer, nasopharyngeal cancer, ovarian cancer, breast cancer, pancreatic cancer and colorectal cancer) and 8 healthy subjects who were undergoing routine health at Dalian Municipal Central Hospital Affiliated of Dalian Medical University. All procedures were performed under the approval of the Local Ethics Committee of the Dalian University of Technology. Different volumes of blood samples can be examined by tailoring the size and quantity of EChry films. A 2-mL (or 4-mL) blood sample was drawn from each patient with advanced (or early-stage cancer). First, the blood samples were treated with red blood cell lysis buffer to remove the red blood cells. The remaining cells in each 2-mL blood were resuspended with 10 mL DMEM, and loaded 1-mL onto 2 cm^2 -EChry film, which has been coated with 1% BSA for 1 h at

room temperature before used. After 60 min of incubation (37 °C, 5% CO_2), the films were washed with PBS for three times. After that, the cells captured on the films were rinsed three times with PBS, fixed with 4% PFA for 15 min, and 1% BSA for 1 h at room temperature. Subsequently, the cells were incubated with a rabbit CD45-polyclonal antibody (10 μg mL^{-1} in PBS) for 30 min at 37 °C. This was followed by another three washes in 1% BSA and further incubation with donkey anti-rabbit antibody (IgG - Alexa Fluor 546 at 2 μg mL^{-1} in PBS) for 30 min at 37 °C. After that, the captured cells were permeabilized with 0.1% Triton X-100 for 10 min, and incubated with Alexa Fluor 488-conjugated pan-cytokeratin monoclonal antibody (AE1/AE3) (anti-CK 5 μg mL^{-1} in PBS) at 37 °C for 30 min. The cells were again washed three times and stained with DAPI (10 μg mL^{-1} in PBS) for 5 min, and then examined and counted under the fluorescent inverted microscope with 10 × objective, which has a broader focusing range to show the overall distribution of CTCs. The captured cancer cells and PBMCs were distinguished from each other based on their fluorescence: blue (DAPI+) and green (CK+) for cancer cells, and blue (DAPI+) and red (CD45+) in the case of PBMCs. The CTC number of 2-mL (or 4-mL) of patients' blood was the sum of captured cells on each EChry film.

Statistical Analysis:

Data are reported as mean ± SD and the statistical significance is determined using one-way ANOVA analysis, which were considered at $p < 0.05$, $p < 0.01$ and $p < 0.001$ levels.

Supporting Text

Preparation of H_2SO_4-etched Pine and Rape Pollens:

The H_2SO_4-etched pine (EPine) and H_2SO_4-etched rape (ERape) pollens were prepared with the same process as for EChry pollens, except that the EChry pollens were replaced with EPine and ERape pollens.

Stability Analysis of pollens on EChry film:

The peeling test was carried out according to previously reported methods. In brief, a piece of 3M Scotch tape (KST1046, adhesive strength higher than 1.23 N cm^{-1}) was pressed down firmly on the EChry film and then quickly removed.[2] The area of pollens remained attached to the EChry film was determined under SEM and reflected light microscope (10 × objective). The images were saved as uncompressed color images (8-bit). The areas covered by the EChry pollen before and after the peeling test were counted using Image-Pro Plus based on the differences in color. The abscission rate of EChry film was determined by the following formula:

$$\text{Abscission rate} = \frac{A_1 - A_2}{A} \times 100\%$$

where, A_1 and A_2 are the areas covered by the EChry pollen before and after the peeling test, respectively. A is the area of the image taken by the microscope (10 × objective).

Preparation of EChry films with different covering-ratios:

EChry pollens were suspended in absolute ethanol to a concentration of 25 mg mL^{-1} and a piece of PVB film was then sprayed with this pollen suspension at a nozzle

height of 10 cm and a carrier gas pressure of 30 psi for 1 ~ 10 s. The EChry film was then characterized using a reflected light microscope (10 × objective), and the images taken were saved as uncompressed color images (JPEG format). The area covered by the EChry pollen (A_{EChry}) was counted using Image-Pro Plus based on the color differences. Then, the ratio of EChry pollen coverage to PVB film was determined by the following formula:

$$\text{Covering-ratio} = \frac{A_{EChry}}{A_{image}} 100\%$$

where A_{EChry} is the area covered by the EChry pollen, and A_{image} is the area of the image (10 × objective) taken by the microscope.

Surface Spray-coating of PS and EChry-bl films:

PS film was prepared under the same condition as for the EChry film, except that the EChry pollens were replaced with PS particles. For the construction of the EChry-bl film, EChry pollens were suspended in 1.5% PVB ethanol solution (v/v), and then sprayed onto the PVB film.

Surface Spray-coating of EPine and ERape films:

EPine and ERape films were prepared under the same condition as for the EChry film, except that the EChry pollens were replaced with EPine and ERape pollens.

Characterization:

X-ray photoelectron spectroscopy (XPS) experiments were performed with an ESCALAB 250Xi X-ray photon-electron spectrometer (Thermo Scientific, U.S.A.) using Mg Kα radiation under a vacuum of 2×10^{-8} Pa. The binding energy (BE) scale

was calibrated by comparing with the neutral adventitious C 1s peak at 284.6 eV. Fourier transform infrared (FTIR) spectrum was measured with a BRUKER-MPA FTIR spectrometer from 4000 to 400 cm^{-1} at room temperature (Bruker, Germany). Fluorescence spectra were recorded on a Fluorescence Spectrophotometer-FP-650 at an excitation wavelength of 405 nm (Jasco, Japan). Roughness (Ra) was measured using a LASER-OLS4000 confocal microscope (Olympus, Japan). The nitrogen adsorption and desorption isotherms were measured at 77 K on a Quantachrome instrument Quadrasor SI. The specific surface area (SSA) was calculated by multi-point BET.

Cell capture experiments performed with EChry-bl, PS, EPine, ERape films and TCPS in culture medium:

EChry-bl, PS, EPine, ERape films and TCPS were coated with 1% BSA before used, and the experimental process was the same as that described for EChry films.

The effective contact area of per filopodia with the nanocage and the bed-of-nails nanostructures:

In the process of filopodia inserting and adhering to the nanocage, the contact area A_{filo} between filopodia and nanocage was estimated as one-second of the area of the flank area of the cylinder,[3] using the following formula:

$$A_{filo} = \frac{\pi d l}{2}$$

where d is the diameter of filopodia was measured as 131 nm and l is the length of the nanocage-inserted filopodia which was measured in TEM images. To ensure that

the filopodia evaluation was statically significant, more than 40 filopodia were used for the evaluation of inserted filopodia length.

For the reported bed-of-nails nanostructures, the contact area (A_{tip}) between the filopodia tip and the nanostructure was considered as the cross section of filopodia tip,[4] calculated using the formula：

$$A_{tip} = \pi(\frac{d}{2})^2$$

where d is the diameter of filopodia which was statistics from the previous reports as 100 ~ 150 nm.[5]

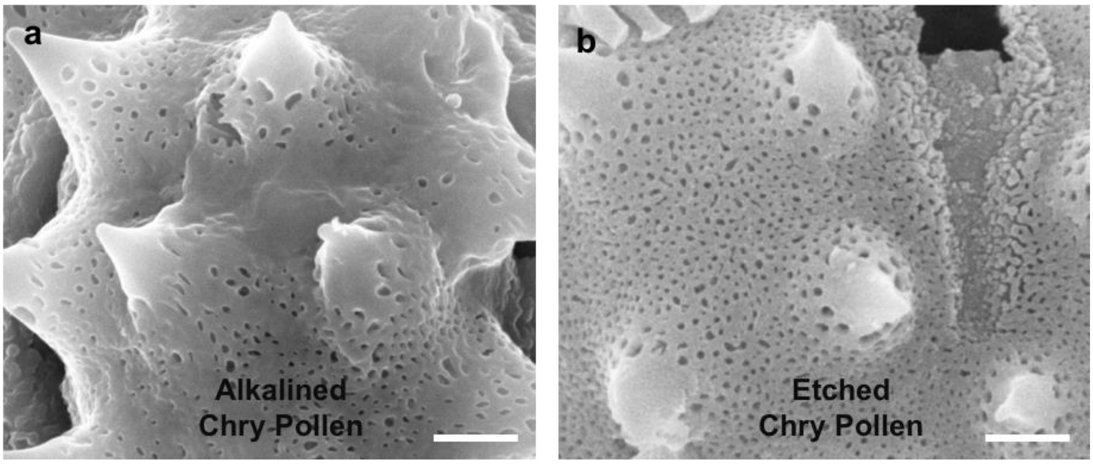

Figure S1. SEM images of nano-scale morphology of a) alkalined Chry pollen and b) H_2SO_4 etched Chry pollen. Scale bar = 5 µm.

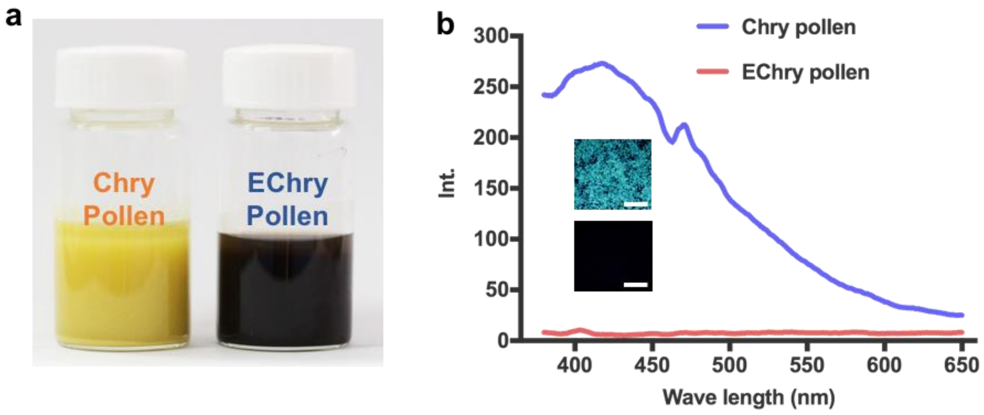

Figure S2. a) Digital photographs of suspensions of native Chry pollens (left) and EChry pollens (right) in glass bottles. b) Wavelength-dependent fluorescent emission spectra of native Chry pollens and EChry pollens at an excitation wavelength of 405 nm. Insets represent fluorescent images of native Chry pollens (up) and EChry pollens (down) at an excitation wavelength of 405 nm. Scale bar = 200 µm.

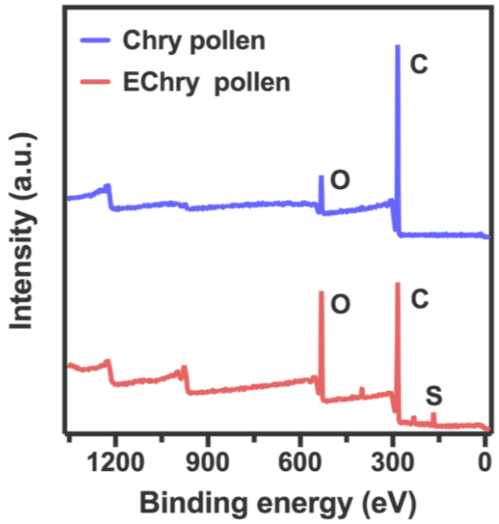

Figure S3. XPS spectra of Chry pollen and EChry pollen.

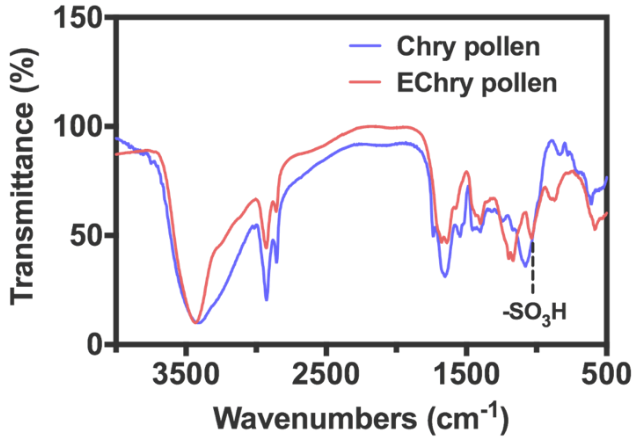

Figure S4. FTIR of Chry pollen and EChry pollen.

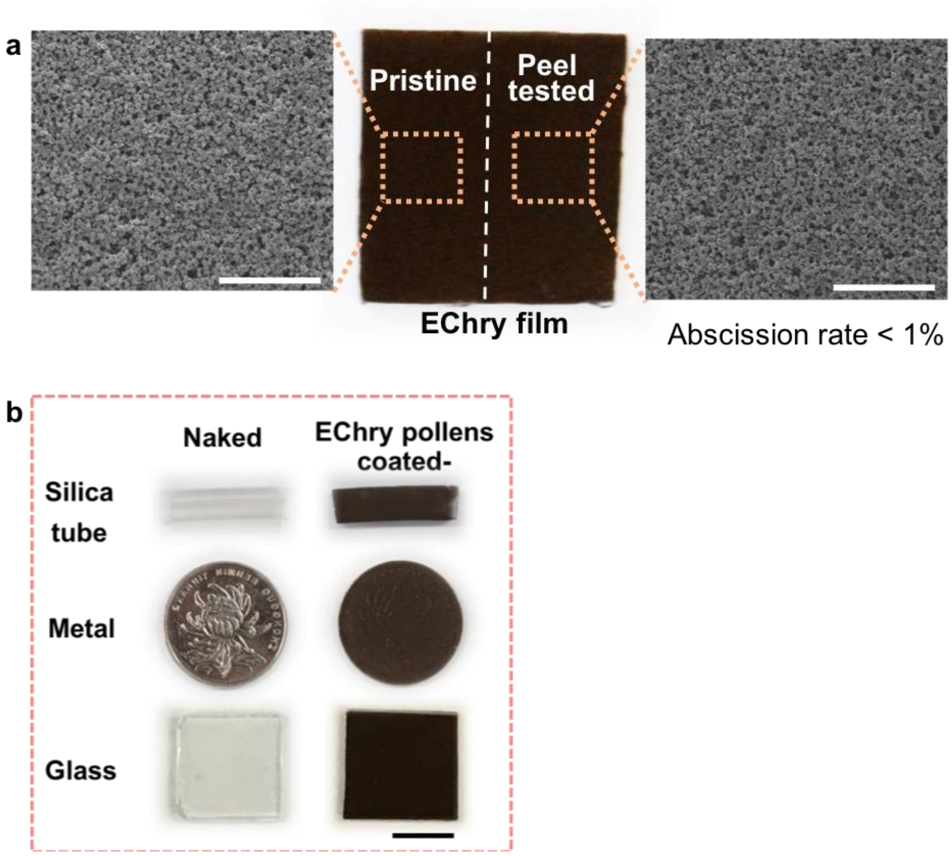

Figure S5. a) SEM images of EChry film before (left) and after (right) the peeling test on EChry film (digital photograph, middle) using a commercial 3M Scotch adhesive tape. Scale bar = 500 µm. b) Digital photographs of naked (left) and EChry pollens-coated (right) silicone tube, metal coin and glass. Scale bar = 10 mm.

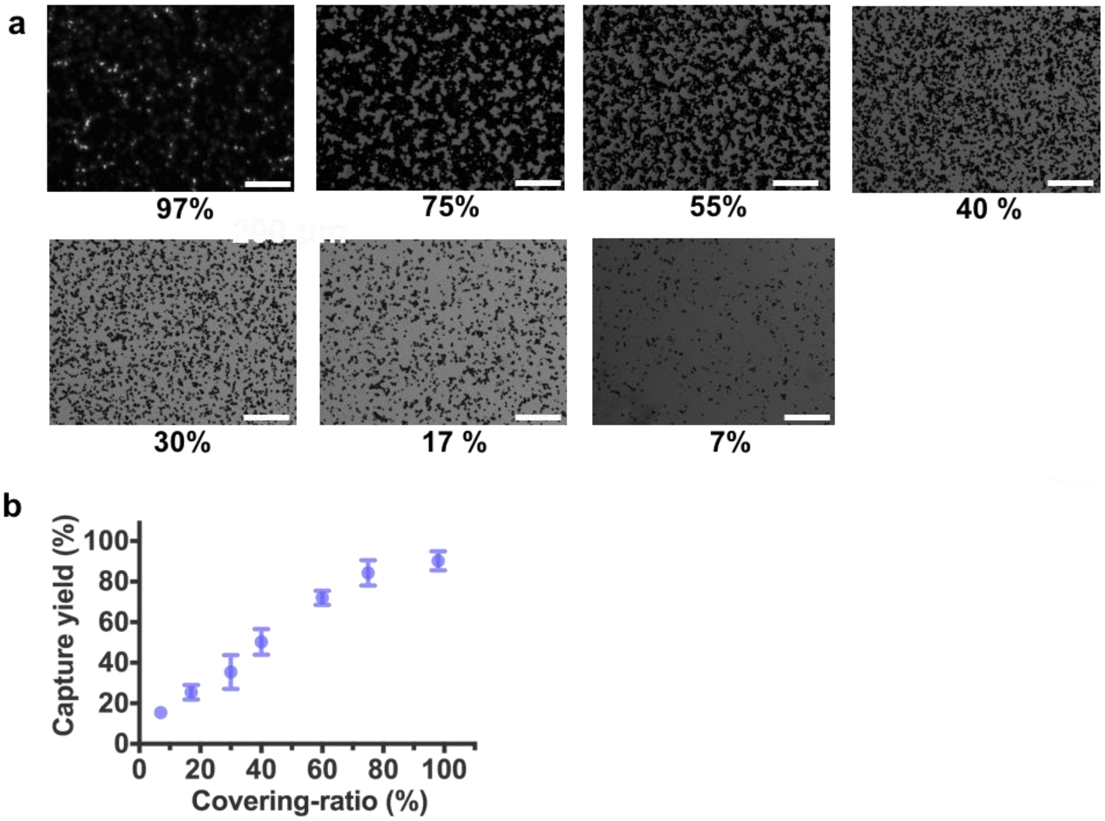

Figure S6. a) Optical microscopy images of EChry films with different covering-ratios. Scale bar = 200 µm. b) Quantitative evaluation of the capture performance of EChry film for MCF-7 cells in culture medium as a function of pollen covering-ratios (mean ± SD, n = 5).

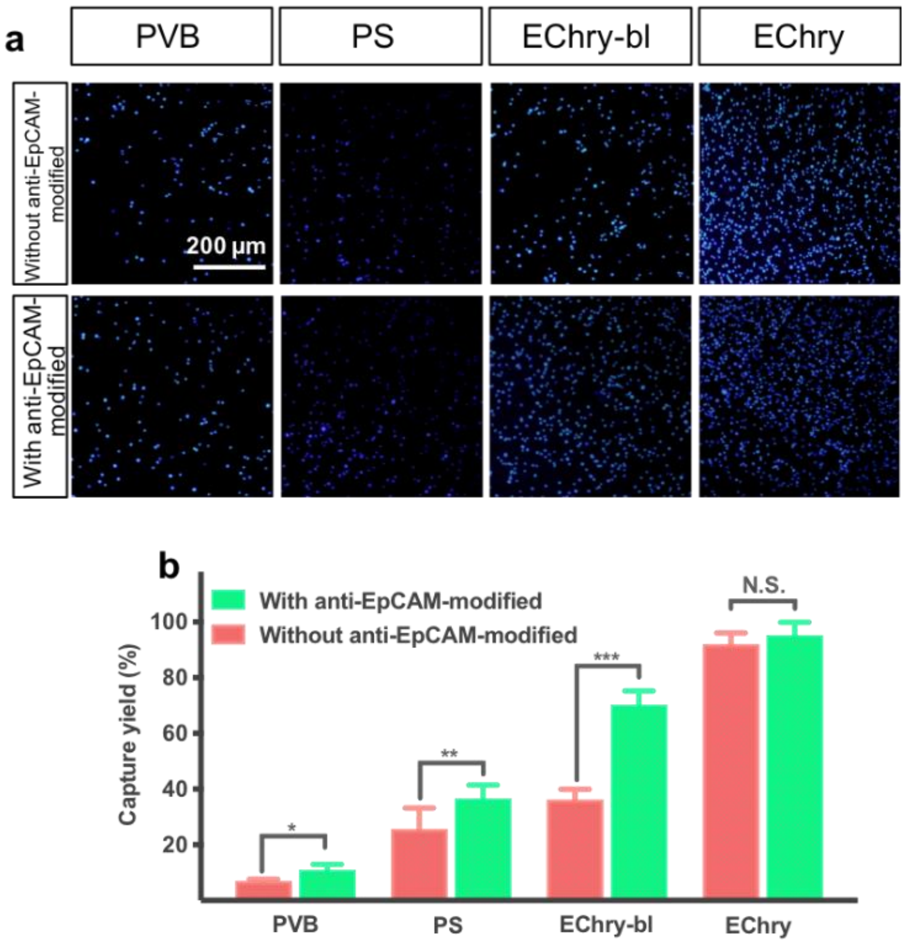

Figure S7. a) Representative fluorescence images of MCF-7 cells captured on the indicated anti-EpCAM-modified and unmodified films. Scale bar = 200 µm. b) Captured yields of MCF-7 cells on the indicated anti-EpCAM modified and unmodified films (mean ± SD, n = 5). *: $p < 0.05$, **: $p < 0.01$, ***: $p < 0.001$. (N.S.) denotes not significant at $p > 0.05$.

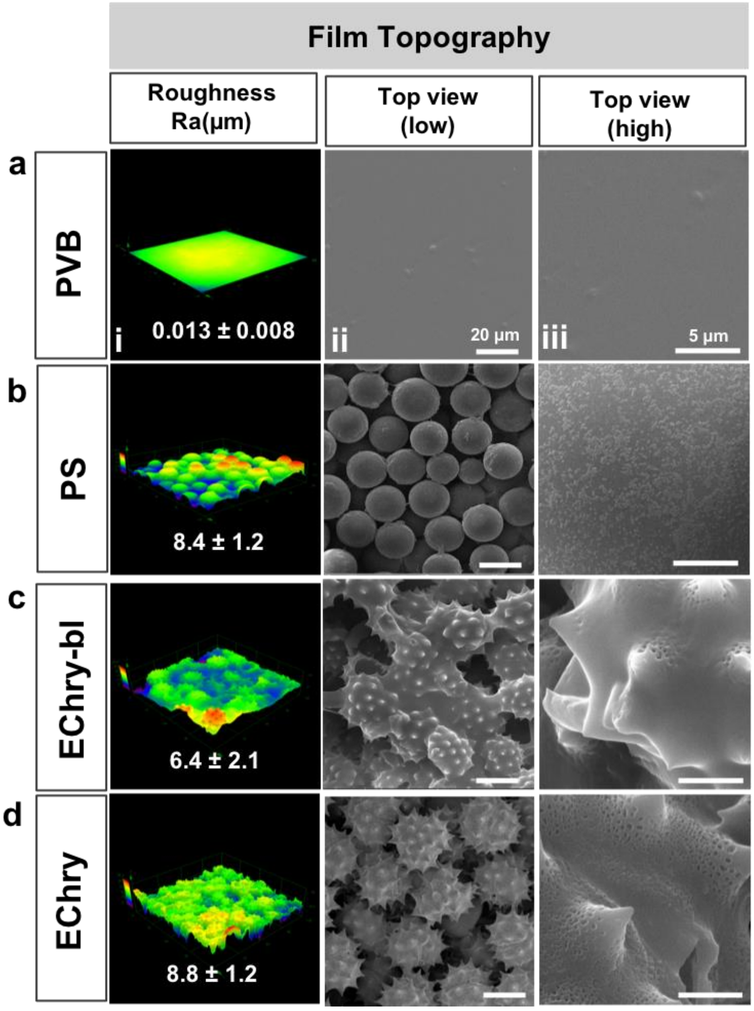

Figure S8. ai-di) CLM topographical images and measured average roughness of PVB, PS, EChry-bl, and EChry films modified on glass substrates. aii-dii) SEM images and aiii-diii) amplifying SEM images of PVB, PS, EChry-bl and EChry films.

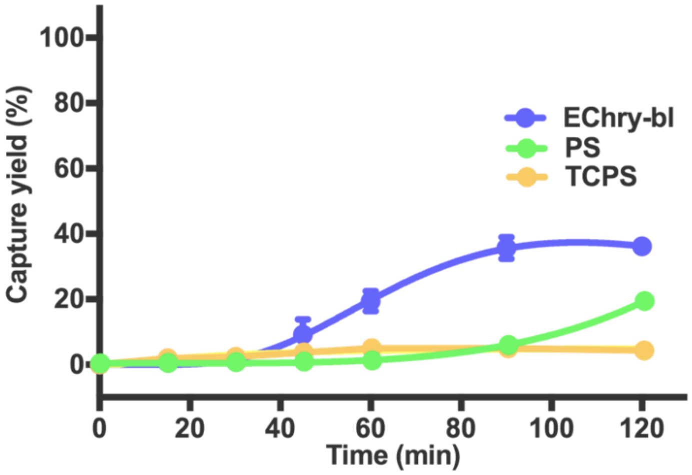

Figure S9. Dynamic capture yields of MCF-7 cells on TCPS, PS and EChry-bl films in culture medium (mean ± SD, n = 5).

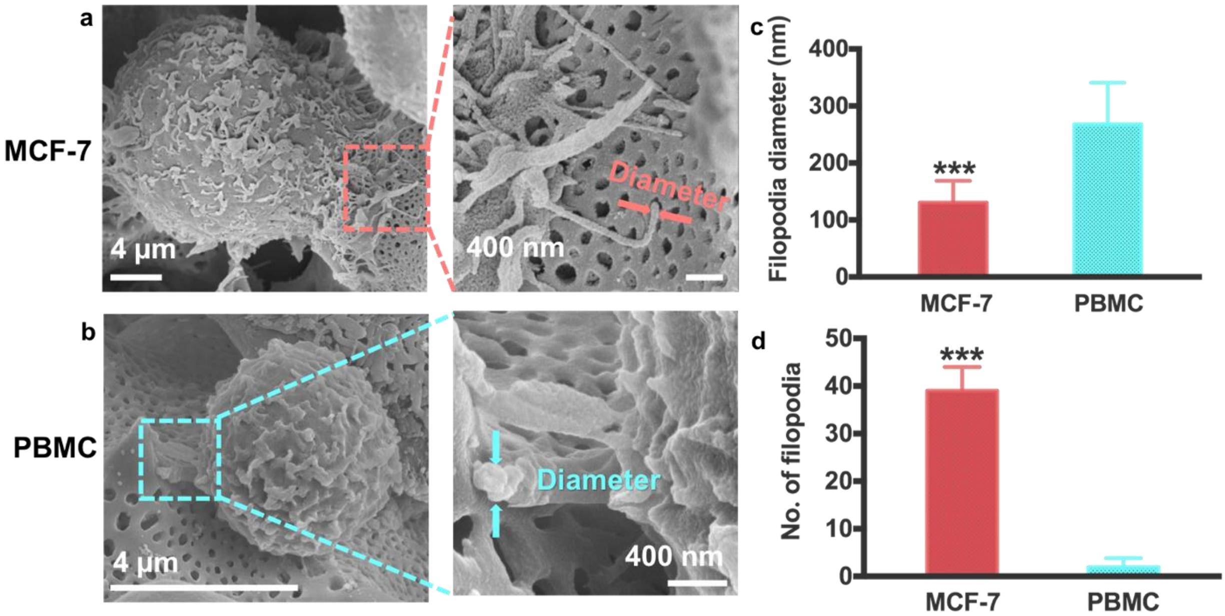

Figure S10. a,b) SEM images of captured MCF-7 cell and adhered PBMC on EChry film (left). Right images are amplifying images of the left corresponding dotted frame. c) The average diameter and d) the average number of protruded filopodia of captured MCF-7 cells and adhered PBMCs. (mean ± SD, n = 30). ***: $p < 0.001$.

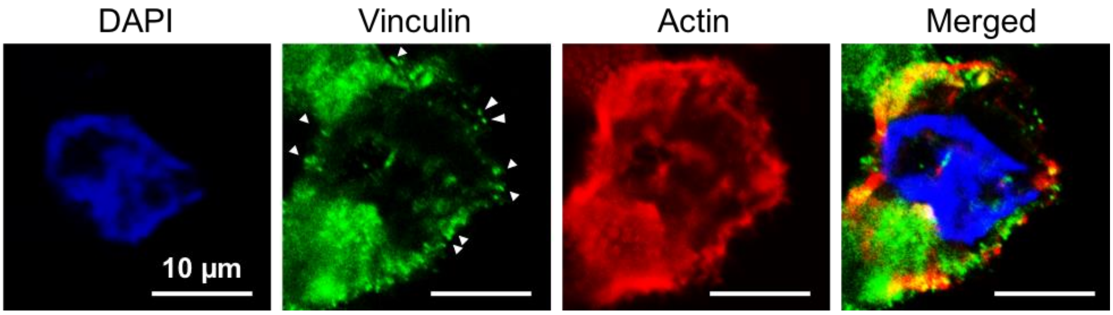

Figure S11. Representative immunofluorescence images of captured MCF-7 cell on the EChry film with 60 min incubation. Cells were co-stained for nuclei (DAPI; blue), vinculin (green) and actin (red). Scale bar = 10 μm. Focal adhesions (FAs) are indicated by the white arrows.

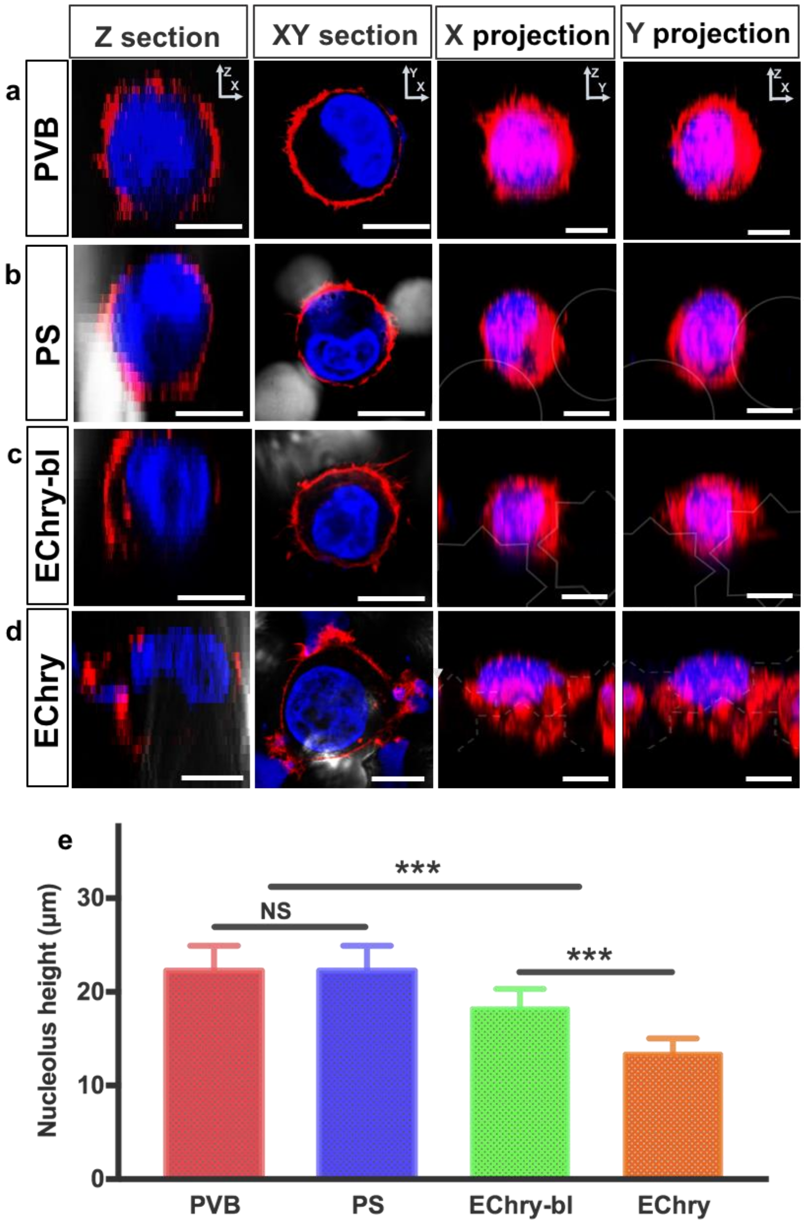

Figure S12. a-d) 3D LSCM fluorescence images (viewed in the reconstructed Z section, cross-sectional XY section, and orthogonal X and Y projection plane) of representative captured MCF-7 cell on EChry, EChry-bl, PS, and PVB films after 60 min cell incubation. The captured cell was costained for the nucleus (DAPI, blue) and actin cytoskeleton (rhodamine, red). Scale bar = 10 μm. PS particles and EChry pollens are outlined in grey. e) Nucleolus height of captured cells (mean ± SD, n = 10). ***: $p < 0.001$. (N.S.) denotes not significant at $p > 0.05$.

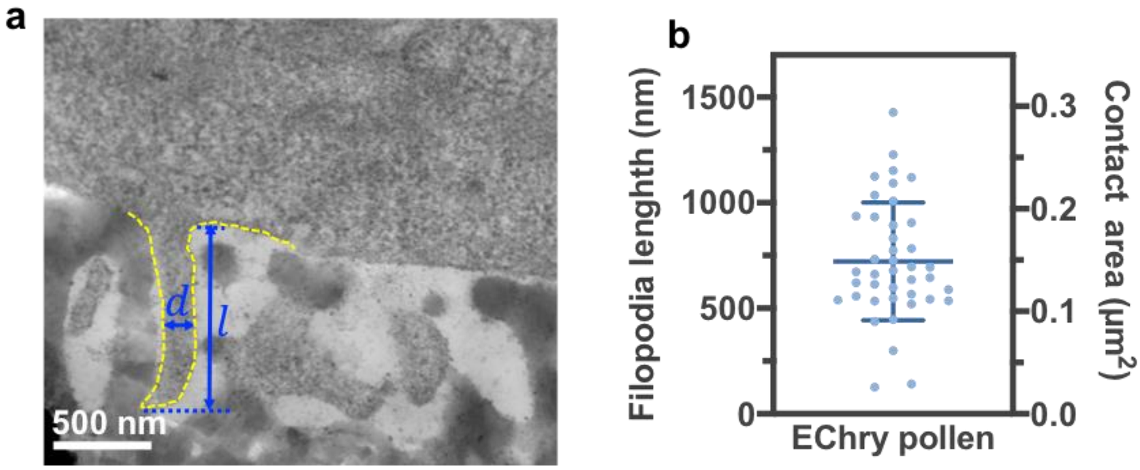

Figure S13. a) Typical TEM image of the filopodia of an MCF-7 cell which inserted into the nanocage cavity. Scale bar = 500 nm. b) The length of the inserted filopodia and the corresponding contact area between the filopodia and nanocage (mean ± SD, n = 41).

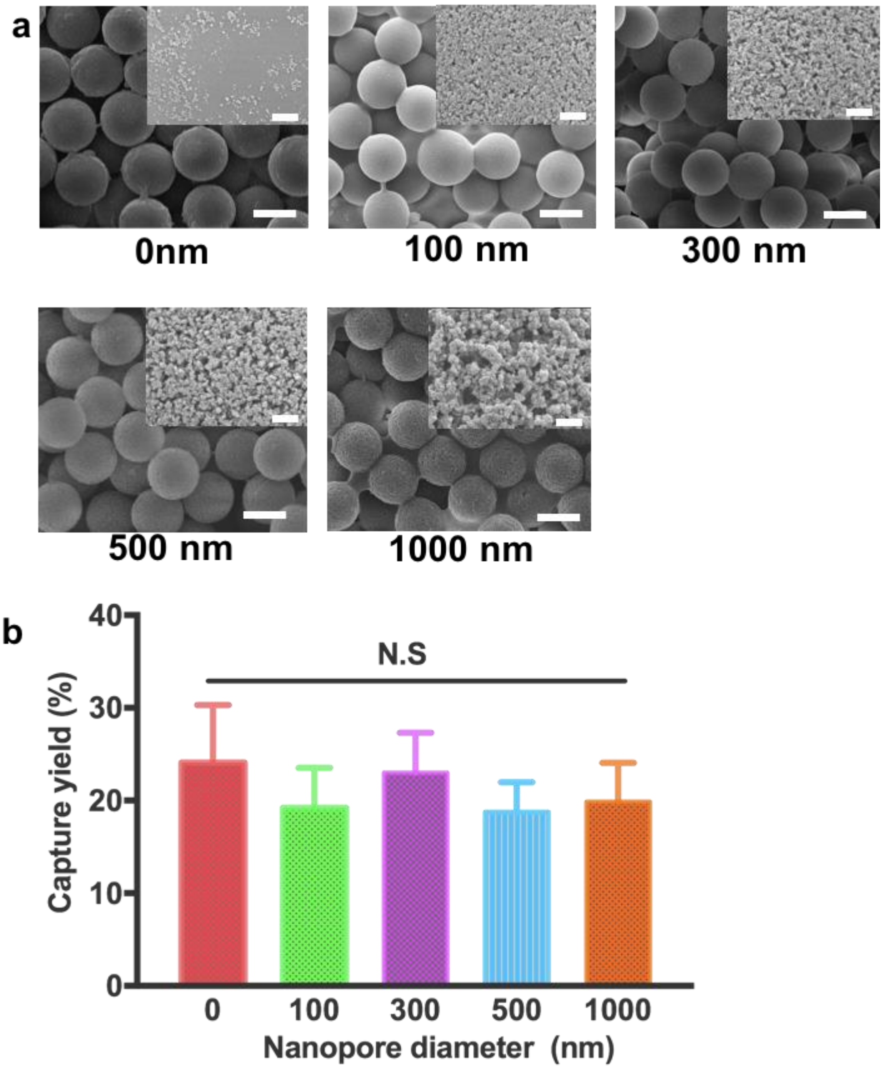

Figure S14. a) SEM images of PS films with indicated nanopore diameter. Scale bar = 20 μm. The insert scale bar = 1 μm. b) Quantitative evaluation of the capture performance of indicated films for MCF-7 cells in culture medium (mean ± SD, n = 5). (N. S.) denotes not significant at $p > 0.05$.

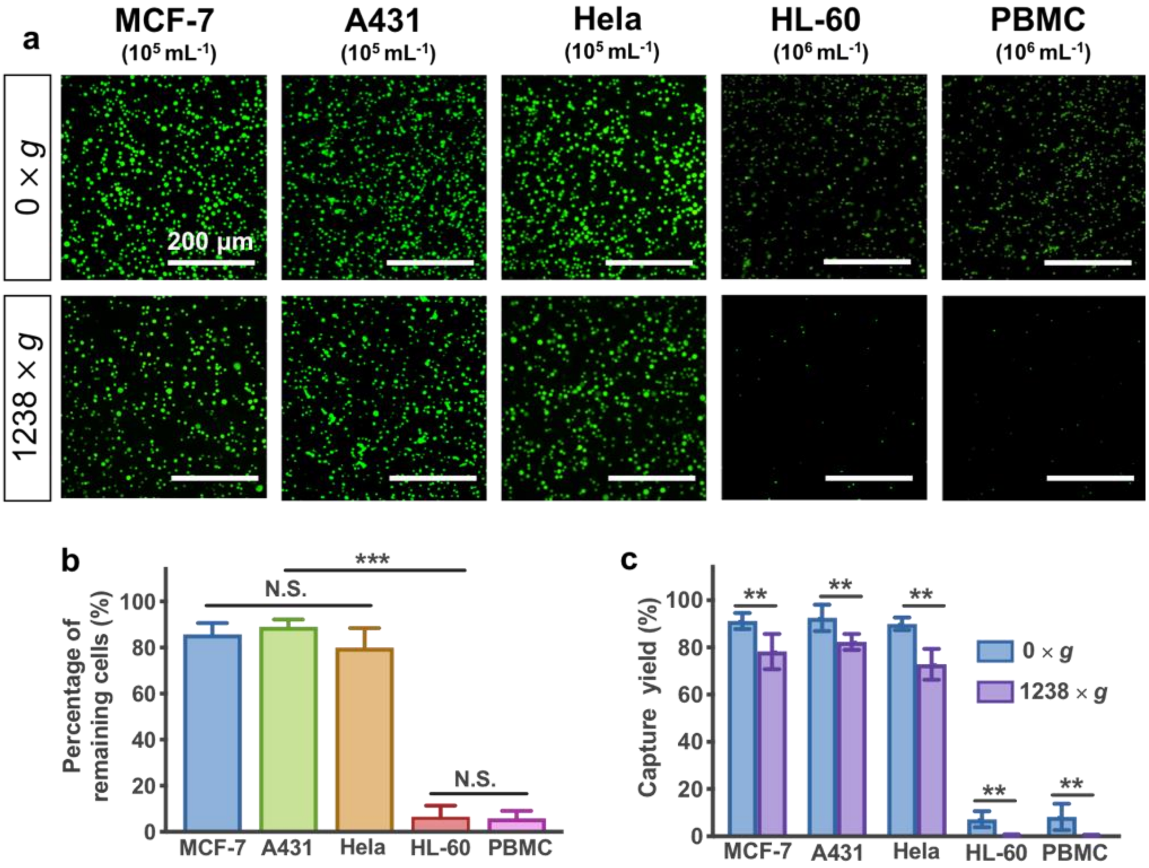

Figure S15. a) Fluorescence images of captured MCF-7, A431, Hela, HL-60 cells and PBMCs on EChry films before and after centrifugation with 1238 × *g*. Scale bar = 200 μm. b) The percentage of remaining cells after centrifugation with 1238 × *g* (mean ± SD, n = 5). (N. S.) denotes not significant at $p > 0.05$. ***: $p < 0.001$. c) Capture yields of indicated cells before and after centrifugation with 1238 × *g* (mean ± SD, n = 5). **: $p < 0.01$.

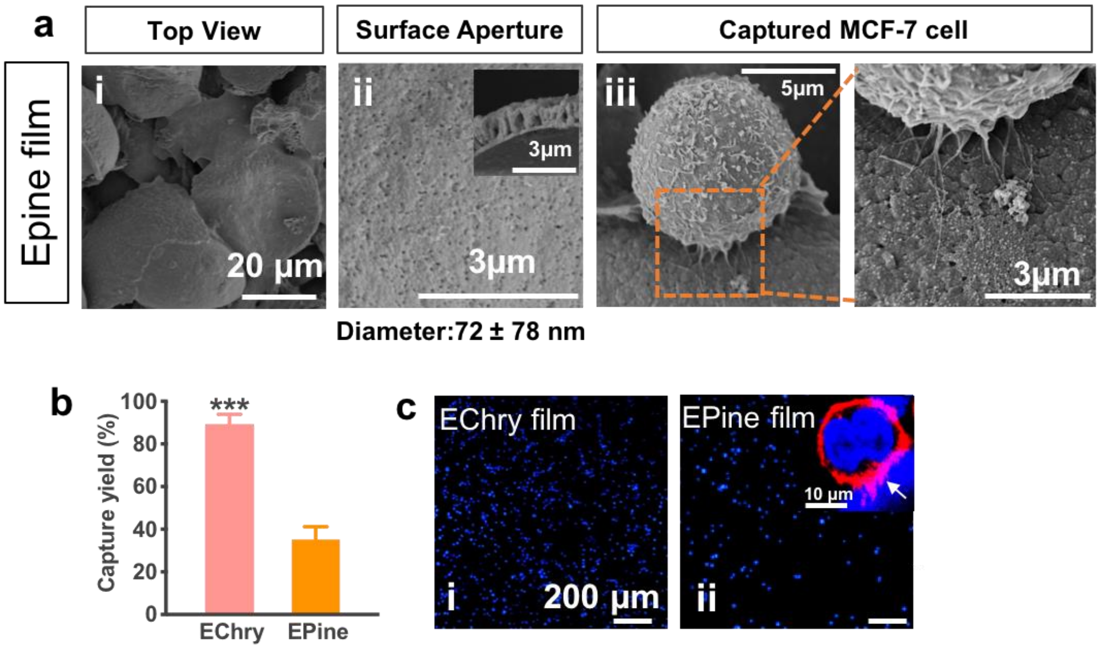

Figure S16. ai) SEM images of EPine film, and aii) amplifying SEM images of the surficial aperture of EPine pollen. The average diameter of the entrance is labeled below. Inset is a cross-sectional SEM image of EPine pollen. aiii) SEM image of a captured MCF-7 cell on EPine film. The right image is an amplifying image of the indicated dotted frame. b) Capture yields of MCF-7 cells on EChry and EPine films (mean ± SD, n = 5). ***: $p < 0.001$. c) Presentative DAPI-stained fluorescence images of MCF-7 cells captured on the indicated films (scale bar = 200 μm). The inset image is the captured cell on EPine pollen, which was costained for the nucleus (DAPI, blue) and actin cytoskeleton (rhodamine, red). The EPine pollen was pointed with a white arrow (scale bar = 10 μm).

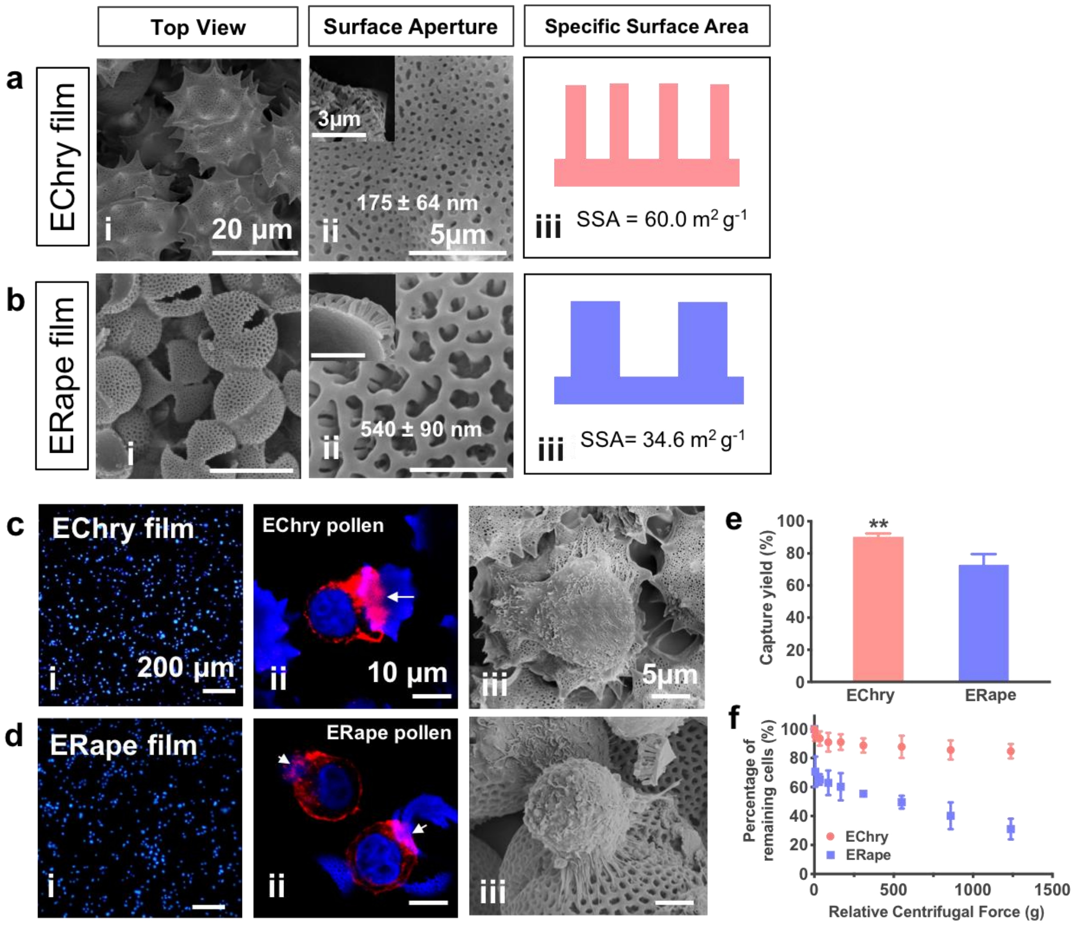

Figure S17. ai-bi) SEM images of EChry and ERape films. aii-bii) Amplifying SEM images of the surficial aperture of EChry and ERape pollen. The average diameter of the entrance is labeled. aiii-biii) Schematic of the effective filopodia-contact area of EChry and Erape pollen. Specific surface area (SSA) is labeled below. ci-di) Presentative DAPI-stained fluorescence images of MCF-7 cells captured on the indicated films. cii-dii) The captured cell was costained for the nucleus (DAPI, blue) and actin cytoskeleton (rhodamine, red). EChry and Erape pollens were pointed with white arrows. ciii-diii) The captured cells on the indicated films. e) Capture yields of MCF-7 cells on EChry and ERape films (mean ± SD, n = 5). **: $p < 0.01$. f) Percentage of the remaining MCF-7 cells on EChry and ERape films under indicated dethatched centrifugal forces (mean ± SD, n = 5)

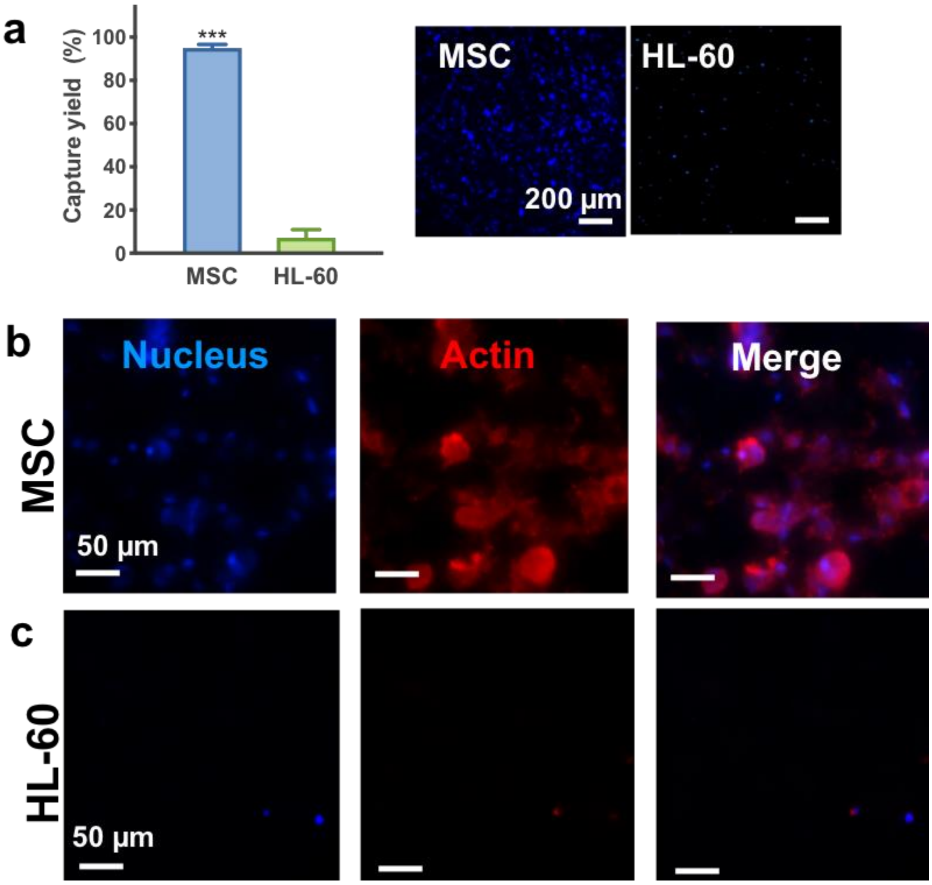

Figure S18. a) Quantitative evaluation of the capture performance of EChry films for 10^5 mL^{-1} MSC and HL-60 cells in the culture medium (left, mean ± SD, n = 5). ***: $p < 0.001$. Presentative DAPI-stained fluorescence images of MSC and HL-60 cells captured on the EChry films (right) (Scale bar = 200 μm). b, c) The captured MSCs and HL-60 cells were costained for the nucleus (DAPI, blue) and actin cytoskeleton (rhodamine, red). Scale bar =50 μm.

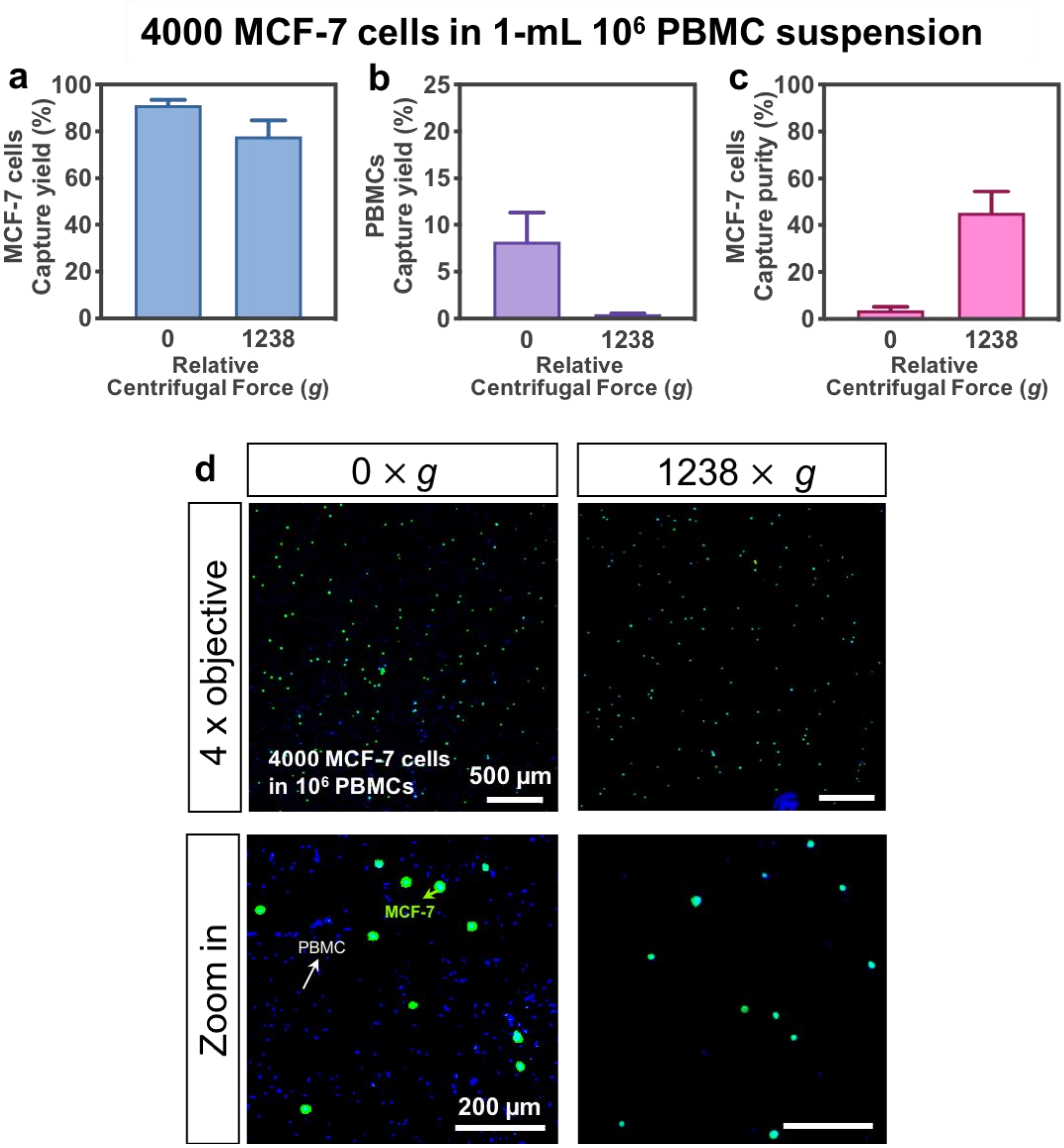

Figure S19. Capture yields and purities with MCF-7 cells. a,b) Capture yields of a) MCF-7 cells and b) PBMCs before and after centrifugation with 1238 × *g* (mean ± SD, n = 5). c) Capture purities of MCF-7 cells on the EChry films before and after centrifugation with 1238 × *g* (mean ± SD, n = 5). d) Captured MCF-7 cells and PBMCs on EChry films before and after centrifugation with 1238 × *g* (mean ± SD, n = 5). The images below are partially enlargements (scale bar = 200 μm).

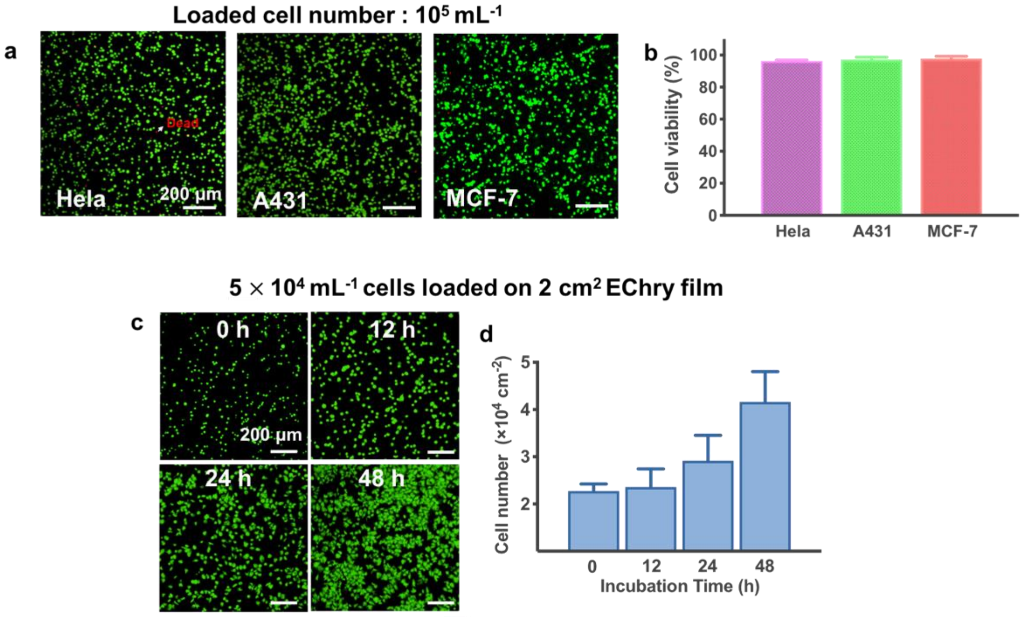

Figure S20. a) Fluorescence images of captured Hela, A431, and MCF-7 cells on EChry films with live/dead staining. Viable cells are shown as green (FDA stain), whereas dead cells are shown as red (PI stain) (scale bar = 200 μm). b) Cell viability of Hela, A431, and MCF-7 cells (mean ± SD, n = 5). c) Fluorescent images of the captured MCF7 cells on EChry films after incubation for 0, 12, 24, and 48 h, respectively (scale bar = 200 μm). d) The number of adherent MCF-7 cells on the EChry films under the indicated culture time (mean ± SD, n = 5).

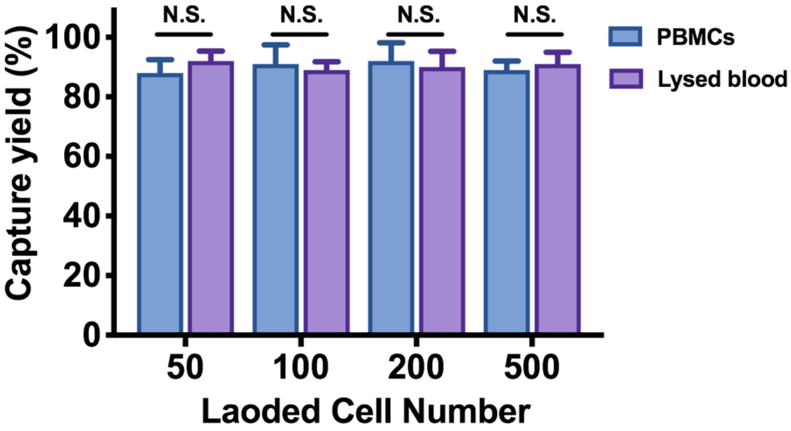

Figure S21. The total number of 50, 100, 200, and 500 MCF-7 cells were loaded into 1 mL of PBMC suspension (10^6 mL^{-1}) and lysed blood sample (diluted by 5 times). Capture yields of rare cancer cells showed no significant difference between the PBMC suspension and diluted lysed blood sample (mean ± SD, n = 5). (N. S.) denotes not significant at $p > 0.05$.

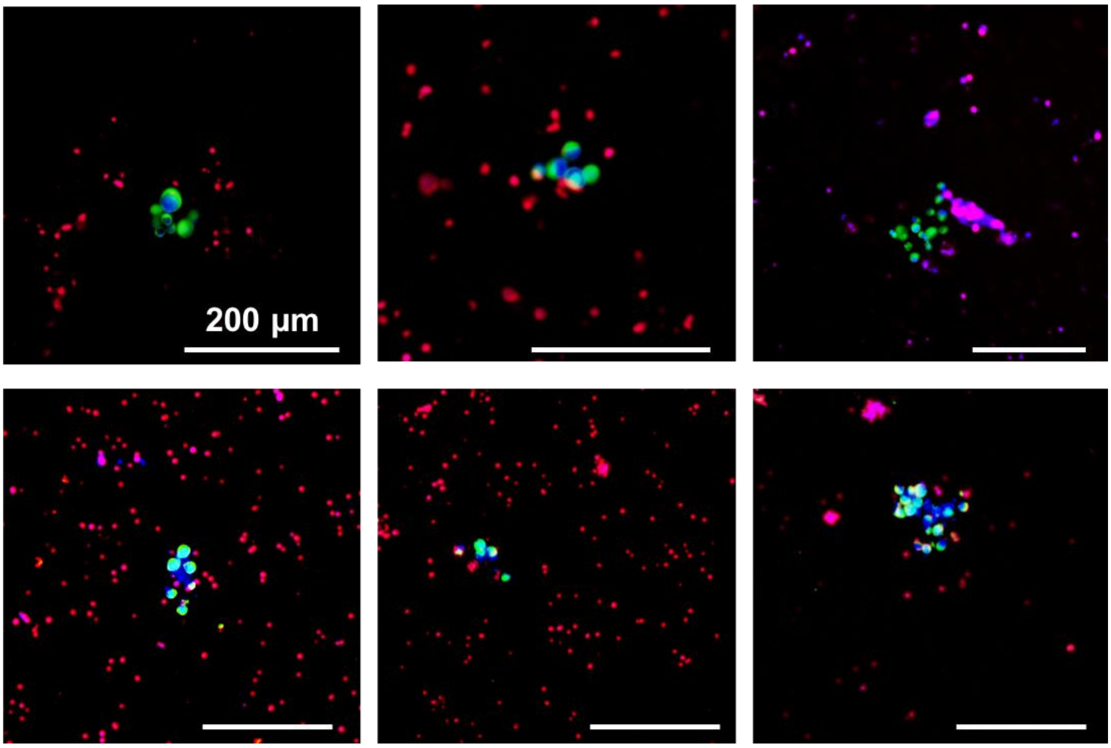

Figure S22. Fluorescence images of the captured CTC clusters from advanced-stage cancer patients on the EChry film. CTC cluster: DAPI+ (blue) and CK+ (green). PBMCs: DAPI+ (blue) and CD45+ (red) (scale bar = 200 µm).

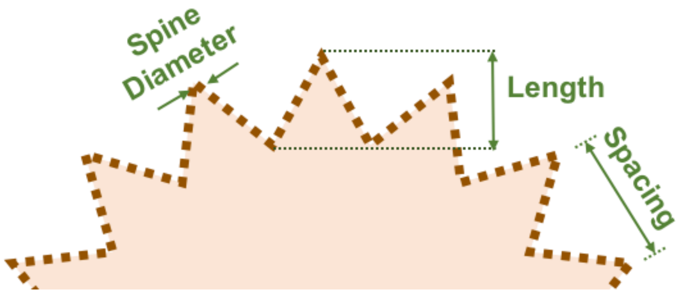

Table S1. Morphological characteristics of EChry pollen.

Structure	Size
Length of spine	3.27 ± 0.43 µm
Spacing between adjacent spine	5.32 ± 0.56 µm
Spine tip-diameter	255 ± 46 nm

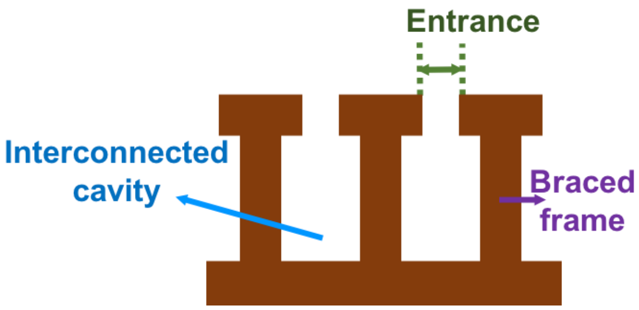

Table S2. Structure characteristics of EChry nanocage

Structure	Size
Nanocage entrance diameter	175 ± 64 nm
Braced frame diameter	117 ± 25 nm
Braced frame height	525 ± 92 nm
Interconnected cavity spacing	166 ± 50 nm

Table S3. Information of advanced-stage cancer patients and the detected CTC and CTC cluster numbers.

Samples No.	Type	Gender	Age	Tumor Stage	CTCs (numbers 2 mL^{-1})	CTC clusters (numbers 2 mL^{-1})
**1**	Lung cancer	F	64	IV	2	0
**2**	Lung cancer	M	49	IV	4	0
**3**	Lung cancer	M	59	IV	3	0
**4**	Lung cancer	F	63	IV	287	17
**5**	Nasopharynx cancer	M	62	IV	2	0
**6**	Laryngeal cancer	M	66	IV	44	8
**7**	Breast cancer	F	60	IV	499	39
**8**	Breast cancer	F	62	IV	197	5
**9**	Celiac cancer	F	63	IV	55	8
**10**	Ovarian cancer	F	59	IV	17	0
**11**	Colon cancer	M	62	IV	373	28

Table S4. Information of early-stage cancer patients and health checkup people and the detected CTC and CTC cluster numbers.

Samples No.	Type	Gender	Age	Tumor Stage	CTCs (numbers 4 mL^{-1})	CTC clusters (numbers 4 mL^{-1})
**1**	Colon cancer	F	42	II	22	3
**2**	Breast cancer	F	53	I	4	0
**3**	Pancreatic cancer	M	80	I	3	0
**4**	Breast cancer	F	30	II	6	0
**5**	Breast cancer	F	70	I	148	9
**6**	Breast cancer	F	39	II	92	1
**7**	Breast cancer	F	56	II	20	0
**8**	Breast cancer	F	46	I	0	0
**9**	Breast cancer	F	59	I	1	1
**10**	Breast cancer	F	55	I	2	0
**1**	Health checkup person	M	25		0	0
**2**	Health checkup person	M	31		0	0
**3**	Health checkup person	F	35		0	0
**4**	Health checkup person	M	30		0	0
**5**	Health checkup person	M	30		0	0
**6**	Health checkup person	F	26		0	0
**7**	Health checkup person	M	26		0	0

References

[1] C. D. Reyes, A. J. Garcia, *J. Biomed. Mater. Res, Part A* **2003**, *67A*, 328.

[2] a) Y. Ha, J. Yang, F. Tao, Q. Wu, Y. Song, H. Wang, X. Zhang, P. Yang, *Adv. Func. Mater.* **2018**, *28*, 1704476; b) J. Ryu, S. H. Ku, H. Lee, C. B. Park, *Adv. Func. Mater.* **2010**, *20*, 2132.

[3] S. Romero, A. Quatela, T. Bornschloegl, S. Guadagnini, P. Bassereau, N. Guy Tran Van, *J. Cell Sci.* **2012**, *125*, 5587.

[4] A. Mogilner, B. Rubinstein, *Biophys. J.* **2005**, *89*, 782.

[5] a) S. Q. Wang, Y. Wan, Y. L. Liu, *Nanoscale* **2014**, *6*, 12482; b) P. C. Zhang, L. Chen, T. L. Xu, H. L. Liu, X. L. Liu, J. X. Meng, G. Yang, L. Jiang, S. T. Wang, *Adv. Mater.* **2013**, *25*, 3566; c) D. J. Kim, G. S. Kim, J. K. Seol, J. H. Hyung, N. W. Park, M. R. Lee, M. K. Lee, R. Fan, S. K. Lee, *J. Biomed. Nanotechnol.* **2014**, *10*, 1030.
